# Impact of Increasing Referral for ^99m^Tc-DPD Scintigraphy on Prognosis Across the Phenotypic Spectrum of Restrictive Cardiomyopathy

**DOI:** 10.1016/j.jacadv.2025.102230

**Published:** 2025-10-22

**Authors:** Chern Hsiang Choy, Chun Shing Kwok, Yasmin Wahid, Boyang Liu, Lauren Turvey-Haigh, Kenneth Parker, James Cullis, Poonam Parekh, Khalid Hussain, Akshanna Akhtar, William M. Bradlow, Alex Zaphiriou, Shanat Baig, Julian D. Gillmore, Marianna Fontana, Jennifer Pinney, Richard P. Steeds, William E. Moody

**Affiliations:** a Midlands Amyloidosis Service, Centre for Rare Diseases, Queen Elizabeth Hospital Birmingham, University Hospitals Birmingham NHS Foundation Trust, Birmingham, United Kingdom; b Institute of Cardiovascular Sciences, College of Medical and Dental Sciences, University of Birmingham, Birmingham, United Kingdom; c Centre for Amyloidosis and Acute Phase Proteins, Division of Medicine, University College London, Royal Free Hospital, London, United Kingdom

**Keywords:** ^99m^Tc-DPD scintigraphy, cardiac amyloidosis, prognosis

## Abstract

**Background:**

Although bone scintigraphy has been widely accepted as integral to the non-biopsy diagnostic algorithm for transthyretin amyloid cardiomyopathy (ATTR-CM), its prognostic role remains uncertain.

**Objectives:**

The authors aimed to characterize changes in the referral pattern for ^99m^Tc-DPD scintigraphy and its influence on the clinical phenotype and prognosis of patients diagnosed with ATTR-CM, and among those individuals in whom ATTR-CM is excluded.

**Methods:**

Retrospective cohort study of all-comers referred for ^99m^Tc-DPD scintigraphy to the Midlands Amyloidosis Service over 15 years.

**Results:**

Of 528 patients referred for ^99m^Tc-DPD scintigraphy, 477 underwent echocardiography suggestive for ATTR-CM and were included in the study. A heightened demand for ^99m^Tc-DPD scintigraphy was linked to increasing proportions of referrals from cardiologists over 5-year periods (47% vs 87% vs 96%, *P* < 0.001). Nearly half of patients (216/477, 45%) were diagnosed with ATTR-CM: 186 had ATTRwt-CM and 30 had ATTRv-CM; the commonest *TTR* variant was V142I (25/30, 83%). Compared to ATTR-CM, patients with nonamyloid CM (261/477, 55%) were younger and more often female yet with similar 5-year mortality rates (40% vs 31%, *P* = 0.044). In an age- and sex-adjusted Cox-proportional hazards model, there was no significant difference in survival between patients with ATTRwt-CM and nonamyloid CM (HR 0.99, 95% CI: 0.98-1.38, *P* = 0.96). There was also no difference in mortality according to Perugini grading.

**Conclusions:**

There is strikingly high mortality among patients with negative ^99m^Tc-DPD scintigraphy, equivalent to that of ATTR-CM. This finding serves as a call for future studies to better characterise this seemingly overlooked cohort, with the aim of developing targeted therapies and improving outcomes.

99mTechnetium labelled 3,3-diphosphono-1,2-propanodicarboxylic acid (99mTc-DPD) scintigraphy has been successfully repurposed as a highly sensitive noninvasive method for imaging transthyretin amyloid cardiomyopathy (ATTR-CM). It plays an integral role within the validated non-biopsy diagnostic algorithm, which has been adopted by international consensus guidelines.^1^ The combination of Perugini grade 2 to 3 uptake, characteristic echocardiography and/or cardiovascular magnetic resonance (CMR) imaging, in the absence of a monoclonal dyscrasia provides >98% diagnostic specificity for ATTR-CM, such that tissue biopsy is no longer required in the majority of cases.^2^ Stratification by Perugini grade of positivity at the time of diagnosis does not, however, appear to confer prognostic significance in ATTR-CM.^3^ Thus, while the diagnostic utility of ^99m^Tc-DPD scintigraphy is undisputed, its role in tracking amyloid burden and informing prognosis in patients with ATTR-CM is still debated.^4^^,^^5^

Restrictive cardiomyopathy (RCM) is a heterogeneous group of diseases characterised by increased myocardial stiffness. Echocardiography demonstrating restrictive pathophysiology in association with increased left ventricular wall thickness should raise a suspicion of cardiac amyloidosis,^6^ often prompting referral for ^99m^Tc-DPD scintigraphy alongside biochemical screening for monoclonal dyscrasia.^7^ While cardiac amyloidosis is now recognised as one of the leading causes of RCM, there are numerous other possible aetiologies. RCM shares significant genotypic and phenotypic overlap with hypertrophic, hypertensive and uraemic cardiomypathies, the cardiac phenotypes evolve over time, and there is frequently confounding related to demographic factors such as ethnicity, obesity and socio-economic status. These factors present a real-world diagnostic challenge for physicians attempting to differentiate diverse aetiologies. This issue has become of critical importance because arriving at the right diagnosis can facilitate the early initiation of appropriate, novel downstream disease-specific therapies.^8^

Thus far, the observational studies of patients referred to specialist amyloidosis centres have focused on subjects with a confirmed diagnosis of amyloidosis.^9^ Very little is known, however, regarding the natural history of those individuals referred with suspected ATTR-CM in whom that diagnosis is excluded. Since 2010, we have been performing ^99m^Tc-DPD scintigraphy in patients referred to the Midlands Amyloidosis Service (MAS) in Birmingham, UK with suspected ATTR-CM. The aims of the present study were to assess: 1) the level of increased demand for 99mTc-DPD scintigraphy in our centre over the past 15 years; and 2) the relative phenotype and prognosis of those in whom ATTR-CM is excluded.

## Methods

### Patient selection

**Figure 1:**
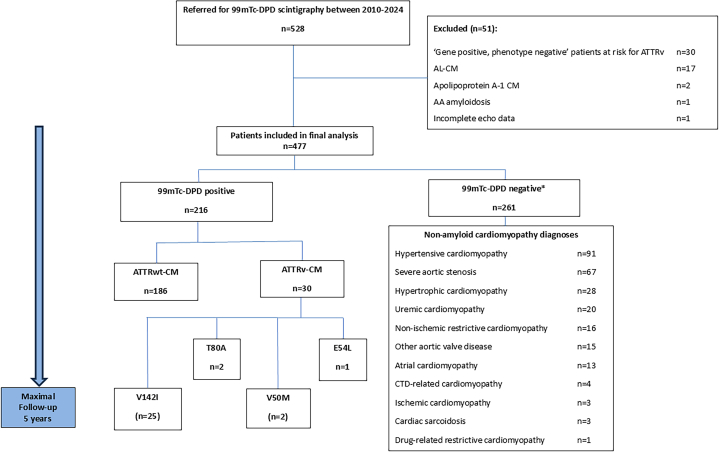
**Study Consort Diagram** ∗One patient with Perugini grade 1 99mTc-DPD scintigraphy, a negative biochemical monoclonal dyscrasia screen, negative genotyping for *TTR* variants and cardiovascular magnetic resonance imaging that was not consistent with cardiac amyloidosis was not thought to have clinically significant ATTR-CM and given a diagnosis of atrial cardiomyopathy as per the consensus of the video multidisciplinary team meeting. The patient remains under close clinical review.

This was a retrospective, single-centre longitudinal cohort study of all patients referred with suspected ATTR-CM to the MAS at Queen Elizabeth Hospital Birmingham. A study consort diagram detailing the identification of patients for inclusion in this study is shown in Figure 1. All patients who underwent ^99m^Tc-DPD scintigraphy between January 2010 and January 2024 were identified. Of 528 patients referred for ^99m^Tc-DPD scintigraphy, 477 had echocardiography studies fulfilling criteria suggestive for cardiac amyloidosis according to expert consensus guidelines,^6^^,^^10^ and were included in the present analysis. We excluded patients in whom the final diagnosis was cardiac AL amyloidosis (n = 17) and other non-TTR forms of cardiac amyloidosis (Apolipoprotein A1, n = 2; AA, n = 1). Patients with ATTRv ‘gene positive’ status under clinical surveillance but without evidence of cardiac amyloidosis (n = 30) were excluded after negative ^99m^Tc-DPD scintigraphy, and one patient was excluded due to incomplete data (Figure 1). Referral practice and the results of ^99m^Tc-DPD were examined over the 15-year study period according to 5-year inclusive intervals: 2010 to 2014, 2015 to 2019 and 2020 to 2024.

This observational cohort study was conducted in accordance with the Declaration of Helsinki and guided by the Strengthening the Reporting of Observational Studies in Epidemiology (STROBE) statement.^11^ Formal ethical approval was not required as the study collated solely clinical, anonymised data and met the criteria for operational improvement activities as per local policy on research conduct.

### Diagnosis of transthyretin amyloid cardiomyopathy

A diagnosis of ATTR-CM was achieved on the basis of characteristic echocardiography or CMR, and Perugini grade 2 to 3 uptake on ^99m^Tc-DPD scintigraphy in the absence of a plasma cell dyscrasia.^2^ Conversely, tissue biopsy was performed in patients with biochemical evidence of a plasma cell dyscrasia despite positive cardiac uptake on ^99m^Tc-DPD scintigraphy to rule out the possibility of cardiac AL amyloidosis. Staging of ATTR-CM was performed at baseline according to N-terminal pro-B-type natriuretic peptide (NT-proBNP) and estimated glomerular filtration rate (eGFR).^12^

### Echocardiography

All transthoracic echocardiograms were performed using PHILIPS EPIQ 7 ultrasound machines. Images were acquired and analysed by British Society of Echocardiography (BSE)-accredited cardiac sonographers in accordance with the joint guidance from the American Society of Echocardiography and the European Association of Cardiovascular Imaging.^13^ From August 2019, echocardiography was performed by a single operator (L.T.) in accordance with BSE guidelines.^6^

### Bone scintigraphy

Patients were administered 600MBq of ^99m^Tc-DPD intravenously before whole-body planar images were acquired 3 hours later using a single-photon emission computed tomography-computed tomography scanner (Siemens Symbia T16, 16 slice; Siemens Symbia Pro-Specta X7, 32 slice). Intensity of myocardial uptake on 99mTc-DPD scintigraphy was graded by two experienced readers (W.E.M, R.P.S.) from 0 to 3 according to the system described by Perugini et al. (ie, score 0, absent cardiac uptake and normal bone uptake; score 1, mild cardiac uptake, inferior to bone uptake; score 2, moderate cardiac uptake accompanied by attenuated bone uptake; score 3, strong cardiac uptake with mild/absent bone uptake).^14^

### Cardiovascular magnetic resonance

Cardiovascular magnetic resonance (CMR) with late gadolinium enhancement (LGE) was performed using a Siemens MAGNETOM Avanto 1.5-T scanner. LGE imaging was acquired using magnitude-only inversion recovery and phase-sensitive inversion recovery sequence reconstructions. T1 mapping was initially carried out using the modified look-locker inversion recovery (MOLLI) sequence to obtain basal, mid and apical ventricular short-axis and 4-chamber long axis images. This was repeated 15 minutes following the intravenous administration of 0.1 mmol/kg of gadobutrol (Gadovist) to produce extracellular volume measurements.^15^ Analysis was performed using cvi42 (version 6.1, Circle Cardiovascular Imaging).

### Diagnosis of Nonamyloid restrictive cardiomyopathies

A diagnosis of cardiac amyloidosis was excluded beyond reasonable doubt in the majority of patients on the basis of a combination of negative ^99m^Tc-DPD scintigraphy, a negative monoclonal dyscrasia screen and negative TTR genotyping. In patients in whom there was still a high index of clinical suspicion for cardiac amyloidosis after clinical review and CMR imaging, a tissue biopsy was performed (usually endomyocardial biopsy). From August 2019, a final diagnosis was attributed to patients after review of all available clinical data and cardiovascular imaging in a UK amyloidosis network video multidisciplinary team meeting involving physicians from the Queen Elizabeth Hospital Birmingham (W.E.M., R.P.S., J.P., S.B.) and the National Amyloidosis Centre (M.F. and J.D.G.), and as part of routine NHS clinical care.^16^ For those cases prior to the inception of the UK amyloidosis network, the diagnosis was assigned after consensus review from 2 clinicians with >10 years experience in cardiovascular imaging and inherited cardiovascular conditions (W.E.M., R.P.S.).

### Statistical analysis

Statistical analysis was performed using Stata (StataCorp. 2013, Stata Statistical Software Release 13, StataCorp LLC, College Station, TX). Categorical data were presented as frequencies and percentages. Continuous variables were tested for normal distribution using the Shapiro-Wilk test and expressed as mean ± SD or median (interquartile range). Means between 2 groups were compared using the Student’s *t*-test if the data were normally distributed; the Mann-Whitney U test was used to evaluate non-parametric data. Differences in means between multiple groups were evaluated using the 1-way ANOVA for parametric data and the Kruskal-Wallis test was used for non-parametric data.

All mortality data were obtained through the UK Office for National Statistics, which is the official government registry for all deaths throughout the United Kingdom. The time to the mortality endpoint was defined as the time from the date of ^99m^Tc-DPD scintigraphy for all deceased patients and for the remainder, time to censor date (April 1, 2024) from the date of ^99m^Tc-DPD scintigraphy. Follow-up was restricted to ≤60 months, after which patients were censored as the majority of events occur in the first 60 months, and a low number of patients are at risk after 60 months.^9^ Kaplan-Meier plots were constructed to estimate survival function from baseline between the different groups according to Perugini grading. Cox proportional hazards regression analysis compared hazard ratios between the 4 grades in the whole cohort, with age and sex as a covariate. The results of the age- and sex-adjusted models were expressed using estimated HRs with their 95% CIs. *P* values <0.05 were considered statistically significant.

## Results

Of the 477 patients identified for inclusion in this study (Figure 1), 216 were diagnosed with ATTR-CM (mean age, 80 ± 7 years; male, 83%), and 261 were diagnosed with a nonamyloid cardiomyopathy (mean age, 73 ± 12 years; male, 68%, *P* < 0.001). Among those diagnosed with ATTR-CM, 186 patients had ATTRwt-CM and 30 had ATTRv-CM. The commonest ATTRv-CM was related to the V142I *TTR* variant (83%).

### Referral practice

**Figure 2:**
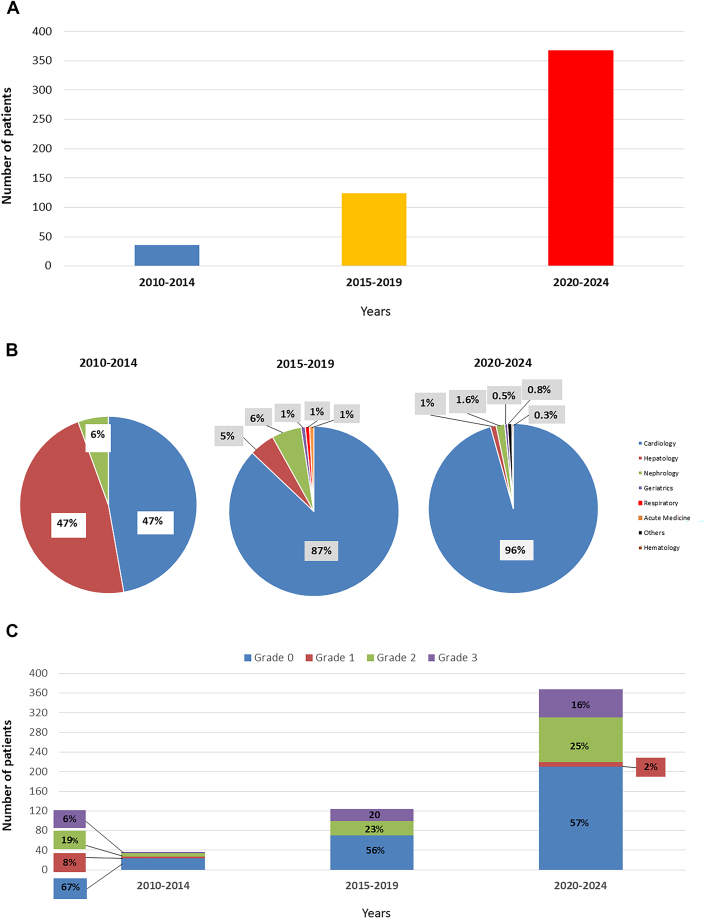
Referral Practice for ^99m^Tc-DPD Scintigraphy Between 2010 and 2024 (A) Number of patients undergoing ^99m^Tc-DPD scintigraphy according to each 5-year time period (n = 528); (B) Number of referrals for ^99m^Tc-DPD scintigraphy according to specialty for each 5-year period (n = 528); (C) ^99m^Tc-DPD scintigraphy results according to each 5-year time period (n = 528).

**Figure 3:**
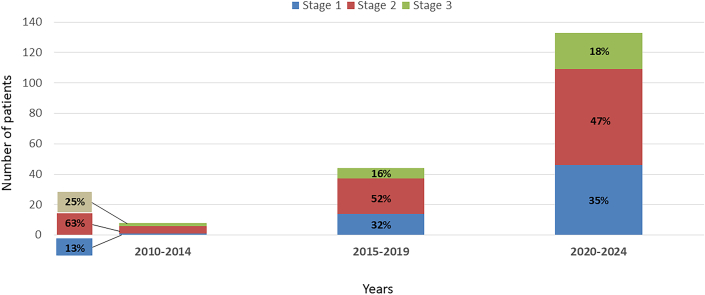
Staging of ATTR-CM Between 2010 and 2024 National Amyloidosis Centre (NAC) staging according to each 5-year time period (n = 185).

The number of referrals for ^99m^Tc-DPD scintigraphy in patients with a differential diagnosis that included ATTR-CM increased for each 5 year period from 36 patients between 2010 to 2014 to 124 patients between 2015 to 2019, and to 368 patients between 2020 to 2024 (Figure 2A). The proportion of patients referred by cardiologists also increased with each 5-year period (47% vs 87% vs 96%, *P* < 0.001) (Figure 2B). While the number of patients referred for assessment increased, the proportion referred and ultimately diagnosed with ATTR-CM did not significantly change over time (Figure 2C). The percentage of patients diagnosed with early-stage ATTR-CM (National Amyloidosis Centre Stage 1) however, did increase over each 5 year interval (13% vs 32% vs 35%, *P* = 0.035) (Figure 3).

### Baseline characteristics at time of diagnosis

The baseline characteristics of the patients included in the analysis are displayed in Table 1. Patients with ATTRwt-CM were the oldest (mean age 81 ± 6 years); in contrast, patients with ATTRv-CM and nonamyloid CM were on average 8 years younger (mean age 73 years). Few women (14%) were diagnosed with ATTRwt-CM, but more were diagnosed in the ATTRv-CM (37%) and nonamyloid CM cohorts (68%). More than three-quarters (77%) of the patients with ATTRv-CM were of Afro-Caribbean ancestry compared with only 21% of the nonamyloid CM and 5% of the ATTRwt-CM cohorts (*P* < 0.001). There were also differences in the frequency of co-morbidities across groups; hypertension and diabetes mellitus were more prevalent in the nonamyloid CM (70% and 31%, respectively). In keeping with this finding, systolic blood pressure was highest in the nonamyloid CM group (*P* = 0.031). Nearly half of patients with ATTR-CM (41%) had a history of carpel tunnel surgery and 10% had a history of lumbar spinal stenosis. There was no significant difference in NYHA class across the groups (*P* = 0.065). Atrial fibrillation was common at baseline across all groups, with a frequency of 70%, 30%, and 43% among those subjects with ATTRwt-CM, ATTRv-CM and nonamyloid CM, respectively. Both the serum NT-proBNP and serum hs-Troponin levels were significantly lower in patients with nonamyloid CM compared with ATTR-CM (both *P* < 0.001). Tissue biopsies (endomyocardial, bone marrow and fat) were more commonly performed in patients with a final diagnosis of ATTR-CM (up to 10% of cases) compared with nonamyloid CM (3% of cases).

**Table 1:** Baseline Characteristics of Patients

	ATTRwt-CM (n = 186)	ATTRv-CM (n = 30)	ATTR-CM (n = 216)	Nonamyloid Cardiomyopathy (n = 261)	*P* Value^a^	*P* Value^b^
Age (y) (±SD)	81 ± 6	73 ± 10	80 ± 7	73 ± 12	<0.001	<0.001
Male, n (%)	160 (86%)	19 (63%)	179 (83%)	177 (68%)	<0.001	<0.001
Ethnicity, n (%)					0.003	<0.001
White	173 (93%)	6 (20%)	179 (83%)	186 (71%)		
Asian	1 (1%)	1 (3%)	2 (1%)	18 (7%)		
Afro-Caribbean	10 (5%)	23 (77%)	33 (15%)	55 (21%)		
Other	2 (1%)	0 (0%)	2 (1%)	2 (1%)		
BMI (kg/m^2^)[IQR]	27 [24-30]	26 [24-29]	27 [24-30]	27 [24-32]	0.48	0.65
Lumbar spinal stenosis, n (%)	18 (10%)	3 (10%)	21 (10%)	5 (2%)	<0.001	0.001
Carpal tunnel syndrome surgery, n (%)	72 (39%)	16 (53%)	88 (41%)	24 (9%)	<0.001	<0.001
Diabetes mellitus, n (%)	33 (18%)	9 (30%)	42 (19%)	80 (31%)	0.003	0.005
Hypertension, n (%)	89 (48%)	17 (57%)	106 (49%)	183 (70%)	<0.001	<0.001
Systolic BP [IQR]	136 [120-153]	130 [117-150]	134 [120-152]	145 [127-161]	0.013	0.031
Diastolic BP (±SD)	75 ± 11	79 ± 11	76 ± 11	77 ± 14	0.18	0.42
Aortic stenosis, n (%)	10 (5%)	0 (0%)	10 (5%)	67 (26%)	<0.001	<0.001
NT-proBNP (ng/L), [IQR]	4,017 [2,036-6,461]	2,508 [1,370-5,725]	3,799 [1,896-6,427]	1,565 [620-5,952]	<0.001	<0.001
hs-Troponin (ng/L) [IQR]	70 [48-116]	113 [58-189]	74 [48-125]	26 [10-81]	<0.001	<0.001
eGFR (mL/min) (±SD)	58 ± 18	59 ± 21	58 ± 19	53 ± 24	0.043	0.12
NAC staging, n (%)					-	0.74
1	51 (27%)	10 (33%)	61 (28%)			
2	80 (43%)	11 (37%)	91 (42%)	-		
3	28 (15%)	5 (17%)	33 (15%)	-		
Missing data	27 (15%)	4 (13%)	31 (14%)	-		
NYHA functional class, n (%)					0.019	0.065
I	19 (10%)	1 (3%)	20 (9%)	33 (13%)		
II	95 (51%)	19 (63%)	114 (53%)	94 (36%)		
III	59 (32%)	10 (33%)	69 (32%)	90 (34%)		
IV	3 (2%)	0 (0%)	3 (1%)	9 (3%)		
Missing data	10 (5%)	0 (0%)	10 (5%)	35 (13%)		
ECG, n (%)					<0.001	<0.001
Sinus rhythm	41 (22%)	19 (63%)	60 (28%)	131 (50%)		
Atrial fibrillation	131 (70%)	9 (30%)	140 (65%)	112 (43%)		
Missing data	14 (8%)	2 (7%)	16 (7%)	18 (7%)		
^99m^Tc-DPD, n (%)					<0.001	<0.001
Grade 0	0 (0%)	0 (0%)	0 (0%)	260 (100%)		
Grade 1	7 (4%)	1 (3%)	8 (4%)	1 (0.4%)^c^		
Grade 2	113 (61%)	11 (37%)	124 (57%)	0 (0%)		
Grade 3	66 (35%)	18 (60%)	84 (39%)	0 (0%)		
Tissue biopsy, n (%)						
Endomyocardial	6 (3%)	0 (0%)	6 (3%)	2 (1%)	0.089	0.10
Bone marrow	10 (5%)	1 (3%)	11 (5%)	3 (1%)	0.011	0.033
Fat	3 (2%)	2 (7%)	5 (2%)	1 (0.4%)	0.060	0.012
Renal	0 (0%)	0 (0%)	0 (0%)	1 (0.4%)	0.36	0.66
No. patients undergoing any biopsy	19 (10%)	3 (10%)	22 (10%)	7 (3%)	0.002	0.008

Values are n (%), mean (±SD), or median [IQR].

a For comparison between ATTR-CM and nonamyloid cardiomyopathy.

b For comparison between ATTRwt-CM, ATTRv-CM and nonamyloid cardiomyopathy.

c One patient with Perugini grade 1.^99m^Tc-DPD scintigraphy, a negative biochemical monoclonal dyscrasia screen and cardiovascular magnetic resonance imaging that was *not* consistent with cardiac amyloidosis was not thought to have clinically significant ATTR-CM and given a diagnosis of atrial cardiomyopathy as per the consensus of the video multidisciplinary team meeting. The patient remains under close clinical review.

### Conventional heart failure and device therapy

A summary of the treatments adminstered to patients is available in Supplemental Table 1. The requirement for anticoagulation was greatest in those patients with ATTRwt-CM (75%). Similarly, there was greater use of digoxin in ATTRwt-CM (25%) compared with ATTRv-CM and nonamyloid CM, which reflects the higher rate of atrial fibrillation in the ATTRwt-CM cohort. Beta-blockers were most commonly prescribed for patients with nonamyloid CM (59%) although half the patients with ATTRwt-CM (49%) were still on these agents at baseline, with the lowest rate of use in ATTRv-CM (27%). Mineralocorticoid receptor antagonists were used in roughly half of all patients with ATTR-CM (47%) with lesser use in non-amyloid CM (25%). Similarly, there was greater use of SGLT2 inhibitors among patients with ATTR-CM (19%) vs nonamyloid-CM (9%). There was no significant difference in the rates of prior pacemaker implantation between groups, with overall roughly 1 in 4 patients having an intra-cardiac device at baseline.

### Disease-modifying therapy

At the time of this study, in the UK the only patients eligible for amyloid specific disease-modifying therapies outside of a trial setting, and approved by the National Institute for Health and Care Excellence were those with hereditary ATTR-polyneuropathy (patisiran and inotersen); a small number of patients were given access to tafamidis from 2019 through an Early Access to Medicines Scheme. Disease-modifying amyloid therapy was given to 9% of patients with ATTRwt-CM and 17% of patients with ATTRv-CM. A total of 6 patients with ATTRv-CM were prescribed patisiran or vutrisiran (small interfering RNA therapy) and 16 patients were administered tafamidis (transthyretin stabiliser).

### Echocardiographic findings

The results of echocardiography are shown in Table 2. Compared to those with ATTR-CM, patients with nonamyloid CM had greater left ventricular (LV) end-diastolic volumes, a higher LV ejection fraction and stroke volume index, and superior myocardial contraction fraction and right ventricular systolic function (defined by tricuspid annular planar systolic excursion). In contrast, patients with ATTR-CM had greater left ventricular wall thickness (IVSd and PWd), left ventricular mass and relative wall thickness. Patients with ATTR-CM also exhibited worse LV diastolic function (as defined by higher E/A ratio, higher septal E/e’ and greater biatrial volumes) compared with the nonamyloid CM cohort.

**Table 2:** Baseline Echocardiography Findings

	ATTRwt-CM (n = 186)	ATTRv-CM (n = 30)	ATTR-CM (n = 216)	NonamyloidCardiomyopathy (n = 261)	*P* Value^a^	*P* Value^b^
Left ventricular ejection fraction (±SD)	49 ± 13	48 ± 17	49 ± 14	53 ± 15	0.008	0.026
IVSd (±SD)	17 ± 3	17 ± 4	17 ± 3	14 ± 4	<0.001	<0.001
GLS (±SD)	−12 ± 4	−10 ± 2	−11 ± 4	−13 ± 4	0.11	0.094
Lateral E/e’ (±SD)	15 ± 5	16 ± 7	15 ± 6	13 ± 6	<0.001	0.003
Septal E/e’ (±SD)	22 ± 9	20 ± 8	21 ± 9	17 ± 8	<0.001	0.002
E/A ratio (±SD)	2.9 ± 7.8	2.1 ± 1.0	2.7 ± 6.7	1.0 ± 0.6	0.005	0.016
Stroke volume indexed (±SD) (mL/m^2^)	23 ± 9	25 ± 7	23 ± 9	28 ± 11	<0.001	<0.001
LVIDd (±SD) (mm)	43 ± 6	42 ± 10	43 ± 7	45 ± 8	0.002	0.008
LVIDs (±SD) (mm)	32 ± 7	31 ± 9	32 ± 7	32 ± 9	0.70	0.77
LVPWd (±SD) (mm)	16 ± 3	16 ± 3	16 ± 3	13 ± 4	<0.001	<0.001
RWT (±SD)	0.8 ± 0.2	0.8 ± 0.3	0.8 ± 0.2	0.6 ± 0.3	<0.001	<0.001
LV mass (±SD) (g)	293 ± 86	302 ± 128	295 ± 93	247 ± 107	<0.001	<0.001
LV mass index (±SD) (g/m^2^)	155 ± 41	165 ± 58	157 ± 44	129 ± 49	<0.001	<0.001
Myocardial volume (±SD) (mL)	282 ± 83	291 ± 124	283 ± 90	237 ± 103	<0.001	<0.001
Myocardial contraction fraction (±SD)	17 ± 10	16 ± 9	17 ± 10	25 ± 13	<0.001	<0.001
LVEDV (±SD) (mL)	90 ± 30	94 ± 41	90 ± 32	102 ± 38	0.008	0.026
LVEDV indexed (±SD) (mL/m^2^)	47 ± 15	51 ± 16	48 ± 15	53 ± 18	0.009	0.021
LVESV (±SD) (mL)	47 ± 22	51 ± 38	48 ± 25	50 ± 31	0.45	0.65
LVESV indexed (±SD) (mL/m^2^)	25 ± 11	26 ± 17	25 ± 12	26 ± 16	0.51	0.75
RA area indexed (±SD) (cm^2^/m^2^)	14 ± 4	12 ± 4	14 ± 4	11 ± 4	<0.001	<0.001
LA volume (±SD) indexed (mL/m^2^)	53 ± 15	48 ± 17	52 ± 15	44 ± 18	<0.001	<0.001
TAPSE (±SD) (mm)	16 ± 5	18 ± 8	17 ± 6	19 ± 6	<0.001	<0.001

Values are mean (±SD).

a For comparison between ATTR-CM and nonamyloid cardiomyopathy.

b For comparison between ATTRwt-CM, ATTRv-CM and nonamyloid cardiomyopathy.

### Cardiac MRI findings

**Figure 4:**
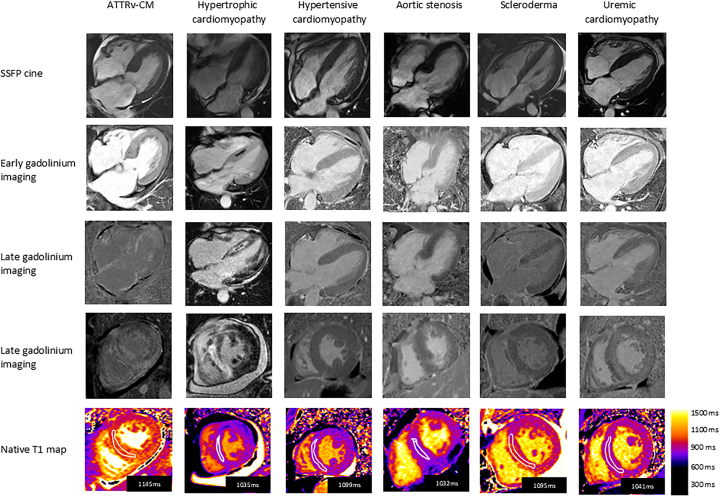
Cardiac Magnetic Resonance Imaging Tissue Characterization Examples of cardiovascular magnetic resonance imaging with tissue characterization to determine aetiology of restrictive cardiomyopathy.

Across the entire study cohort study, a CMR was performed in 322 out of 471 patients (68%). The majority of nonamyloid CM patients (183 of 261, 70%) were assigned a final diagnosis after undergoing CMR. Examples of CMR tissue characterisation to determine the underlying aetiology of RCM are depicted in Figure 4.

### Tissue diagnosis

A tissue biopsy (fat, bone marrow or endomyocardial) was more commonly performed in patients ultimately diagnosed with ATTR-CM (22 out of 216, 10%) than in those with non-amyloid CM (7 out of 261, 3%; *P* < 0.001; Table 1). Compared with fat and bone marrow trephine biopsies, endomyocardial biopsy had the highest diagnostic yield. Immunohistochemistry confirmed the presence of ATTR cardiac amyloid in all patients undergoing endomyocardial biopsy (6 out of 6, 100%) that had characteristic echocardiography and/or CMR for amyloidosis and Perugini grade 2 to 3 ^99m^Tc-DPD scintigraphy and a concomitant paraproteinaemia (monoclonal gammopathy of unknown significance).

### Nonamyloid restrictive cardiomyopathy

Table 3 highlights differences over the 15 year study period (2010-2024) in the baseline characteristics of the patients referred and diagnosed with non-amyloid cardiomyopathy according to 5-year intervals. Patients with nonamyloid CM were being diagnosed at progressively older ages across the 5-year tertiles of year of diagnosis (*P* = 0.024). A male predominance among those diagnosed with non-amyloid CM persisted across the entire study duration (circa. 68%), although this was not to the same extent of that seen among the ATTRwt-CM cohort, in whom 86% were male. Those patients more recently diagnosed with nonamyloid CM tended to have lower baseline levels of hs-Troponin and higher eGFR. Based on echocardiography, patients with a more recent diagnosis of nonamyloid CM had lower left ventricular end-systolic volumes in association with a higher LV ejection fraction. Despite being older at baseline, more contemporaneous non-amyloid CM patients exhibited less adverse remodelling at baseline with lesser degrees of elevated LV mass and left atrial dilatation.

**Table 3:** Baseline Characteristics for patients With Nonamyloid Cardiomyopathy Diagnosed Between 2010 and 2024 According to Year of Diagnosis (N = 261)

	2010-2014 (n = 14)	2015-2019 (n = 59)	2019-2024 (n = 188)	*P* Value
Age, y	68 ± 13	70 ± 14	74 ± 12	0.024
Male, n (%)	9 (64%)	28 (47%)	140 (74%)	0.87
Ethnicity, n (%)				0.25
White	7 (50%)	38 (64%)	141 (75%)	
Asian	2 (14%)	4 (7%)	12 (6%)	
Afro-Caribbean	5 (36%)	17 (29%)	33 (18%)	
Other	0 (0%)	0 (0%)	2 (1%)	
BMI (kg/m^2^)[IQR]	28 [24-31]	26 [23-32]	27 [24-31]	0.67
Lumbar spinal stenosis, n (%)	0 (0%)	0 (0%)	5 (3%)	0.36
Carpal tunnel syndrome surgery, n (%)	0 (0%)	4 (7%)	20 (11%)	0.29
Diabetes mellitus, n (%)	5 (36%)	19 (32%)	56 (30%)	0.92
Hypertension, n (%)	9 (64%)	49 (83%)	125 (66%)	0.17
Systolic BP [IQR]	145 [127-161]	147 [127-161]	145 [127-160]	0.84
Diastolic BP (±SD)	77 ± 15	79 ± 14	77 ± 14	0.68
NT-proBNP (ng/L), [IQR]	5,049 [1,188-12,008]	1,691 [651-6,182]	1,397 [607-5,653]	0.37
hs-Troponin (ng/L) [IQR]	43 [36-81]	34 [21-109]	20 [9-69]	0.054
eGFR (mL/min) (±SD)	46 ± 17	49 ± 23	56 ± 24	0.11
NYHA functional class, n (%)				0.51
I	1 (7%)	7 (12%)	25 (13%)	
II	8 (57%)	20 (34%)	66 (35%)	
III	3 (21%)	26 (44%)	61 (32%)	
IV	1 (7%)	1 (2%)	7 (4%)	
Missing data	1 (7%)	5 (8%)	29 (15%)	
ECG, n (%)				0.22
Sinus rhythm	4 (29%)	31 (53%)	96 (51%)	
Atrial fibrillation	9 (64%)	24 (41%)	79 (42%)	
Missing data	1 (7%)	4 (7%)	13 (7%)	
Anticoagulation, n	10 (71%)	26 (44%)	79 (42%)	0.10
Beta-blockers, n (%)	12 (86%)	36 (61%)	105 (56%)	0.092
ACEI/Entresto, n (%)	10 (71%)	35 (59%)	93 (49%)	0.15
MRA, n (%)	6 (43%)	17 (29%)	41 (22%)	0.14
Digoxin, n (%)	4 (29%)	10 (17%)	28 (15%)	0.40
SGLT2 inhibitor, n (%)	1 (7%)	3 (5%)	19 (10%)	0.48
Device, n (%)				0.12
None	10 (71%)	49 (83%)	135 (72%)	
VVI/DDD PPM	0 (0%)	4 (7%)	29 (15%)	
CRT	2 (14%)	0 (0%)	8 (4%)	
ICD	1 (7%)	3 (5%)	7 (4%)	
ILR	0 (0%)	1 (2%)	2 (1%)	
Missing data	1 (7%)	2 (3%)	7 (4%)	
Left ventricular ejection fraction (±SD)	42 ± 17	54 ± 15	53 ± 14	0.033
IVSd (±SD)	15 ± 4	15 ± 4	14 ± 3	0.27
GLS (±SD)	-	−16 ± 0	−13 ± 4	0.45
Lateral E/e’ (±SD)	13 ± 5	14 ± 6	12 ± 6	0.27
Septal E/e’ (±SD)	17 ± 2	19 ± 8	17 ± 8	0.67
E/A ratio (±SD)	2.0 ± 1.3	1.0 ± 0.5	1.0 ± 0.5	<0.001
Stroke volume indexed (±SD) (mL/m^2^)	29 ± 11	28 ± 12	28 ± 10	0.91
LVIDd (±SD) (mm)	49 ± 7	44 ± 10	45 ± 8	0.17
LVIDs (±SD) (mm)	40 ± 9	32 ± 9	31 ± 8	0.011
LVPWd (±SD) (mm)	13 ± 5	13 ± 5	12 ± 3	0.12
RWT (±SD)	0.6 ± 0.2	0.7 ± 0.5	0.6 ± 0.1	0.027
LV mass (±SD) (g)	301 ± 145	255 ± 121	239 ± 96	0.12
LV mass index (±SD) (g/m^2^)	155 ± 71	135 ± 59	125 ± 43	0.081
Myocardial volume (±SD) (mL)	290 ± 139	245 ± 117	230 ± 93	0.12
Myocardial contraction fraction (±SD)	26 ± 14	23 ± 12	25 ± 13	0.67
LVEDV (±SD) (mL)	132 ± 41	101 ± 45	99 ± 33	0.056
LVEDV indexed (±SD) (mL/m^2^)	69 ± 20	53 ± 23	52 ± 15	0.047
LVESV (±SD) (mL)	76 ± 40	50 ± 34	48 ± 29	0.048
LVESV indexed (±SD) (mL/m^2^)	39 ± 20	26 ± 17	25 ± 15	0.067
RA area indexed (±SD) (cm^2^/m^2^)	13 ± 4	11 ± 4	11 ± 4	0.14
LA volume (±SD) indexed (mL/m^2^)	50 ± 14	49 ± 24	41 ± 16	0.028
TAPSE (±SD) (mm)	16 ± 6	19 ± 5	20 ± 6	0.056

Values are (%), mean (±SD), or median [IQR].

For the 261 patients in whom cardiac amyloidosis was excluded, the most frequent alternative diagnoses included hypertensive cardiomyopathy (n = 91), severe aortic stenosis (n = 67), hypertrophic cardiomyopathy with restrictive physiology (n = 28), and uraemic cardiomyopathy (n = 20) (Table 4).

**Table 4:** Baseline Characteristics for Patients With Nonamyloid Cardiomyopathy Diagnosed According to Aetiology

	Hypertensive Cardiomyopathy (n = 91)	Aortic Stenosis (n = 67)	Hypertrophic Cardiomyopathy (n = 28)	Uraemic Cardiomyopathy (n = 20)	*P* Value
Age (±SD) (y)	70 ± 12	81 ± 8	68 ± 14	65 ± 16	<0.001
Male, n (%)	65 (71%)	44 (66%)	21 (75%)	17 (85%)	0.38
Ethnicity, n (%)					0.001
White	51 (56%)	61 (91%)	20 (71%)	13 (65%)	
Asian	7 (8%)	4 (6%)	0 (0%)	2 (10%)	
Afro-Caribbean	31 (34%)	2 (3%)	8 (29%)	5 (25%)	
Other	2 (2%)	0 (0%)	0 (0%)	0 (0%)	
BMI [IQR] (kg/m^2^)	27 [25-31]	26 [23-32]	26 [23-29]	25 [22-34]	0.63
Lumbar spinal stenosis, n (%)	3 (3%)	2 (3%)	0 (0%)	0 (0%)	0.67
Carpal tunnel syndrome surgery, n (%)	10 (11%)	6 (9%)	1 (4%)	1 (5%)	0.60
Diabetes mellitus, n (%)	39 (43%)	15 (22%)	7 (25%)	8 (40%)	0.019
Hypertension, n (%)	84 (92%)	39 (58%)	16 (57%)	15 (75%)	<0.001
Systolic BP [IQR]	150 [129-160]	145 [127-162]	138 [132-154]	144 [122-165]	0.020
Diastolic BP (±SD)	81 ± 16	75 ± 12	76 ± 14	75 ± 15	0.11
NT-proBNP (ng/L), [IQR]	1,380 [440-6,067]	1,446 [617-4,663]	1,734 [338-9,675]	7,587 [1,062-30,874]	0.50
hs-Troponin (ng/L) [IQR]	26 [16-81]	20 [8-128]	40 [18-93]	59 [19-154]	0.26
eGFR (mL/min) (±SD)	54 ± 22	58 ± 22	52 ± 22	19 ± 20	<0.001
NYHA functional class, n (%)					0.48
I	12 (13%)	9 (13%)	4 (14%)	1 (5%)	
II	35 (38%)	24 (36%)	13 (46%)	8 (40%)	
III	30 (33%)	28 (42%)	9 (32%)	6 (30%)	
IV	1 (1%)	4 (6%)	0 (0%)	2 (10%)	
Missing data	13 (14%)	2 (3%)	2 (7%)	3 (15%)	
ECG, n (%)					0.41
Sinus rhythm	46 (51%)	36 (54%)	12 (43%)	12 (60%)	
AF	39 (43%)	30 (45%)	15 (54%)	5 (25%)	
Missing data	6 (7%)	1 (1%)	1 (4%)	3 (15%)	
Biopsy, n (%)					
Endomyocardial	0 (0%)	0 (0%)	1 (4%)	0 (0%)	0.094
Bone marrow	2 (2%)	0 (0%)	0 (0%)	1 (5%)	0.32
Fat	0 (0%)	0 (0%)	0 (0%)	1 (5%)	0.025
Renal	0 (0%)	0 (0%)	1 (4%)	0 (0%)	0.094
Anticoagulation, n (%)	39 (43%)	35 (52%)	15 (54%)	5 (25%)	0.13
Beta-blockers, n (%)	49 (54%)	46 (69%)	19 (68%)	13 (65%)	0.23
ACEi/Entresto, n (%)	62 (68%)	30 (45%)	14 (50%)	8 (40%)	0.010
MRA, n (%)	30 (33%)	10 (15%)	8 (29%)	3 (15%)	0.046
Digoxin, n (%)	15 (16%)	7 (10%)	6 (21%)	2 (10%)	0.46
SGLT2 inhibitor, n (%)	13 (14%)	3 (4%)	4 (14%)	1 (5%)	0.16
Device, n (%)					0.042
None	68 (75%)	56 (84%)	16 (57%)	15 (75%)	
VVI/DDD PPM	9 (10%)	8 (12%)	3 (11%)	4 (20%)	
CRT	3 (3%)	3 (4%)	1 (4%)	1 (5%)	
ICD	5 (5%)	0 (0%)	4 (14%)	0 (0%)	
ILR	1 (1%)	0 (0%)	2 (7%)	0 (0%)	
Missing data	5 (5%)	0 (0%)	2 (7%)	0 (0%)	
Left ventricular ejection fraction (±SD)	49 ± 17	55 ± 14	59 ± 14	55 ± 8	0.026
IVSd (±SD) (mm)	15 ± 4	14 ± 3	18 ± 4	14 ± 4	<0.001
GLS (±SD) (%)	−12 ± 4	-	−12 ± 3	-	0.81
Lateral E/e’ (±SD)	13 ± 5	14 ± 7	13 ± 5	13 ± 4	0.83
Septal E/e’ (±SD)	16 ± 6	18 ± 9	18 ± 9	19 ± 7	0.70
E/A ratio (±SD)	1.1 ± 0.7	0.9 ± 0.4	1.0 ± 0.6	1.1 ± 0.3	0.29
Stroke volume indexed (±SD) (mL/m^2^)	26 ± 10	29 ± 11	26 ± 8	35 ± 14	0.087
LVIDd (±SD) (mm)	46 ± 9	45 ± 6	41 ± 8	47 ± 10	0.031
LVIDs (±SD) (mm)	32 ± 10	31 ± 7	28 ± 8	34 ± 6	0.19
LVPWd (±SD) (mm)	13 ± 3	12 ± 2	14 ± 4	15 ± 7	0.004
RWT (±SD)	0.6 ± 0.2	0.6 ± 0.1	0.8 ± 0.3	0.8 ± 0.8	0.003
LV mass (±SD) (g)	273 ± 116	222 ± 81	281 ± 139	286 ± 110	0.025
LV mass index (±SD) (g/m^2^)	143 ± 55	119 ± 39	141 ± 63	142 ± 45	0.041
Myocardial volume (±SD) (mL)	262 ± 112	214 ± 78	271 ± 133	275 ± 106	0.025
Myocardial contraction fraction (±SD)	20 ± 9	26 ± 10	24 ± 12	27 ± 12	0.023
LVEDV (±SD) (mL)	108 ± 39	91 ± 30	92 ± 31	130 ± 41	0.009
LVEDV indexed (±SD) (mL/m^2^)	57 ± 19	47 ± 13	45 ± 15	67 ± 23	0.001
LVESV (±SD) (mL)	59 ± 37	38 ± 21	39 ± 23	62 ± 20	0.006
LVESV indexed (±SD) (mL/m^2^)	31 ± 19	20 ± 12	19 ± 11	32 ± 10	0.004
RA area indexed (±SD) (cm^2^/m^2^)	11 ± 4	11 ± 4	11 ± 3	12 ± 5	0.87
LA volume (±SD) indexed (mL/m^2^)	43 ± 20	45 ± 17	43 ± 15	48 ± 17	0.77
TAPSE (±SD) (mm)	19 ± 5	20 ± 5	18 ± 5	23 ± 4	0.015

Values are n (%), mean (±SD), or median [IQR].

Severe aortic stenosis (n = 67), hypertrophic cardiomyopathy with restrictive physiology (n = 28), and uraemic cardiomyopathy (n = 20).

### All-cause mortality

**Figure 5:**
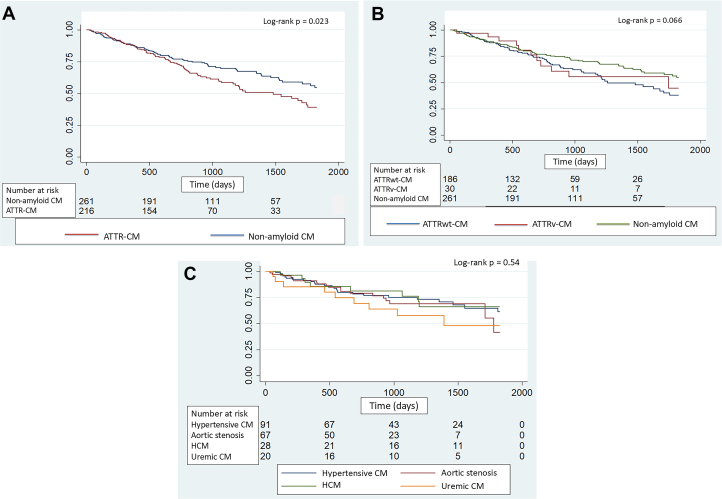
Unadjusted Kaplan-Meier Analysis of Survival According to Disease Cohort (A) All-cause mortality in patients with ATTR-CM and nonamyloid cardiomyopathy, (B) All-cause mortality in patients with ATTRwt-CM, ATTRv-CM and nonamyloid cardiomyopathy, and (C) All-cause mortality in patients with nonamyloid cardiomyopathy according to diagnosis.

The unadjusted Kaplan-Meier 5 year survival curves for the amyloid CM and nonamyloid CM cohorts are illustrated in Figures 5A to 5B. The 5-year mortality rate in patients with ATTR-CM was 40% (87/216) compared to 31% (82/261) in patients with nonamyloid CM (*P* = 0.044). In a Cox-proportional hazards model after adjustment for age and sex, there was no difference in survival between patients with ATTRwt-CM and nonamyloid CM (HR: 0.99, 95% CI: 0.98-1.38, *P* = 0.96) and ATTRv-CM (HR: 1.30, 95% CI: 0.68-2.49, *P* = 0.42). In keeping with these results, among all-comers, there was also no statistically significant difference in 5-year mortality rates according to the Perugini grade of positivity (grade 0: 31% (82/261), grade 1: 12.0% (1/8), grade 2: 42% (52/124) and grade 3: 41% (34/84), *P* = 0.26).

In a sub-group analysis of mortality among those patients with nonamyloid cardiomyopathy, although uraemic cardiomyopathy was associated with the highest mortality rate (45% mortality after a median 2.7-year follow-up) (Supplemental Table 2), there was no significant difference in mortality between the 4 most prevalent nonamyloid diagnoses (log rank *P* = 0.54) (Figure 5C).

## Discussion

This study has highlighted increasing referral for ^99m^Tc-DPD imaging over the last 15 years, in particular from cardiologists, that has contributed to earlier diagnosis of ATTR-CM according to NAC staging. Nearly half of all patients referred for ^99m^Tc-DPD scintigraphy are subsequently diagnosed with ATTR-CM, the majority with ATTRwt-CM. Consistent with previous data,^3^^,^^17^ Perugini grade of positivity at baseline does not appear to confer prognostic significance in patients with established ATTR-CM but our study has also uncovered a high mortality in those patients referred for ^99m^Tc-DPD scintigraphy with nonamyloid CM, despite being younger and more often female (Central Illustration).

**Central Illustration:**
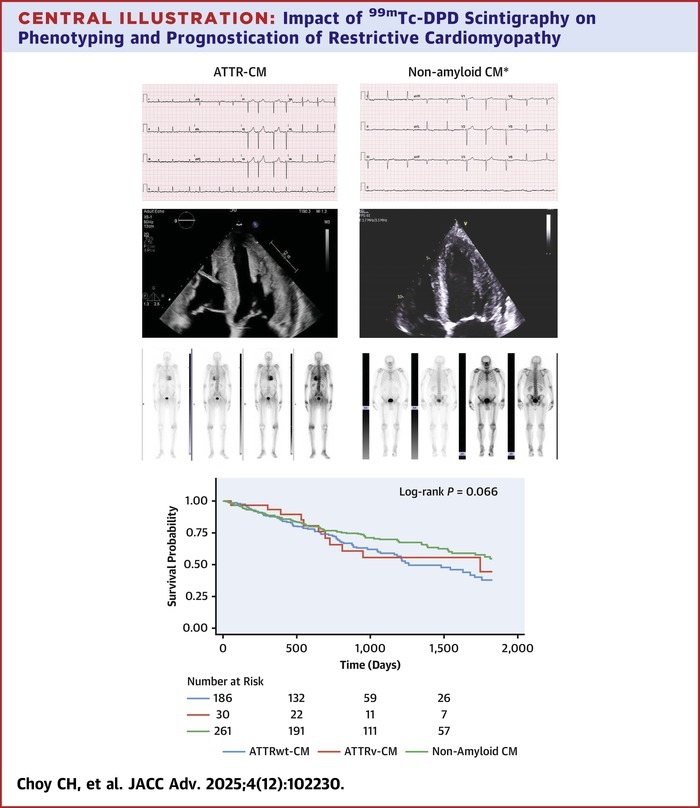
**Impact of ^99m^Tc-DPD Scintigraphy on Phenotyping and Prognostication of Restrictive Cardiomyopathy** ∗ECG and imaging in this case is taken from a patient diagnosed with uraemic cardiomyopathy.

In keeping with previous reports^9^^,^^18^ from the UK and Italy, this study has demonstrated a surge in the number of referrals for ^99m^Tc-DPD scintigraphy following the incorporation of the non-biopsy diagnostic algorithm into international consensus guidelines.^1^^,^^2^^,^^10^ A greater awareness of ATTR-CM, fuelled by the emergence of disease modifying therapies has driven increasing rates of referral, particularly among cardiologists. While referrals in 2010 to 2014 for ^99m^Tc-DPD scinitgraphy very often came from hepatologists and nephrologists, those made in the last 10 years were almost exclusively from cardiologists with expertise in heart failure, cardiomyopathy and imaging. This observation reflects a change in the attitude towards this disease. Before the availability of novel TTR directed therapies, there had been understandable reluctance from cardiologists to send patients for ^99m^Tc-DPD scintigraphy; the general perception had been that making a diagnosis of ATTR-CM was unlikely to positively influence patient management or outcomes. A transformation in mindset has led to increasing referral rates over the last decade with our centre seeing a progressive increase in diagnoses, such that ATTR-CM has become one of the more prevalent cardiomyopathy diagnoses. Moreover, the clinical characteristics of patients with ATTR-CM is opportunely changing with patients being diagnosed at earlier NAC stages of the disease, when patients are likely to benefit most from novel therapies. In future, it will be important to ensure there is adequate resource for continued access to early testing with ^99m^Tc-DPD scintigraphy if we are to match the expected growth in ATTRwt-CM among an ageing population.

This study has revealed a growing cohort of patients with nonamyloid forms of RCM emerging as a consequence of the increased interest in diagnosing ATTR-CM (just over half of all those referred to our centre for ^99m^Tc-DPD scintigraphy). This group has an equally poor age-adjusted mortality rate as those diagnosed with ATTR-CM, with a 5-year survival that approaches 50%. To place this finding into context, based on general population UK data from the Office for National Statistics, taking the mean age at diagnosis of nonamyloid CM in the present study, a 73 year old would on average expect to live for a further 14 years if male, and a further 15 years if female.^19^ Contextualizing this finding further, unadjusted mortality rates at 4-years in patients across all levels of untreated aortic stenosis (AS) severity range from 25% in patients with mild AS increasing to 45% among those with severe AS.^20^ A report including patients from the Framingham Heart Study and the Cardiovascular Health Study, suggests that patients with heart failure with preserved ejection fraction (HFpEF) suffer an even higher mortality burden, with 5-year survival rate as low as 36%.^21^ The relative excess mortality associated with a restrictive, non-amyloid CM phenotype uncovered in the present study calls for an urgent need to better characterise this heterogeneous population, in order to identify better targeted and more efficacious treatments.

Important nationwide registry work is now underway with the aim of developing novel approaches to treat heart failure with preserved ejection fraction, which is considered the largest unmet need in cardiovascular medicine.^22^^,^^23^ The ongoing studies of cardiac myosin inhibitors in HCM provide some optimism. In a phase 2 trial, treatment with mavacamten was associated with reduced NTproBNP and hs-Troponin in non-obstructive HCM.^24^ Similarly, an open-label trial of mavacamten in patients with left ventricular hypertrophy and symptomatic HFpEF (defined as a NYHA functional class II or III, LVEF ≥60% and elevation in NT-proBNP at baseline), has also demonstrated reductions in the same biomarkers of cardiac wall stress and injury.^25^ Among patients with mildly reduced or heart failure and preserved ejection fraction (HFpEF) both SGLT2 inhibitors and more recently, steroidal mineralocorticoid receptor antagonists (MRAs) have been shown to reduce the rate of hospitalization for heart failure.^26^^,^^27^ In our study, the rates of prescription of MRAs and SGLT2 inhibitors were notably lower in patients with nonamyloid CM compared to those with ATTR-CM, a potential area which could be targeted in future.

It is notable that a significant proportion of patients in the negative ^99m^Tc-DPD cohort had concomitant aortic stenosis (67 out of 261, 26%). This likely reflects a lower threshold to refer these patients driven by the evidence that older patients with severe aortic stenosis are frequently identified as having ATTRwt-CM.^28^ In contrast, our frequency of severe aortic stenosis and/or history of transcatheter aortic valve replacement (TAVR) in those patients with positive ^99m^Tc-DPD scintigraphy subsequently diagnosed with ATTRwt-CM was low at only 5% (10 out of 186). This rate is less than half of that reported previously but this discrepancy is unlikely to be explained by gender differences between our studies. The current study demonstrated a strong male predominance in ATTRwt-CM; in contrast, Nitsche et al. reported an 11% frequency of positive DPD scintigraphy among patients referred for TAVR despite their cohort having an equal male:female split. This could also reflect referral bias adding support for the notion that we are currently underdiagnosing ATTR-CM in women, with a much higher rate of referral for ^99m^Tc-DPD scintigraphy in males in the present study.

The finding that less than half the patients referred for ^99m^Tc-DPD scintigraphy had a subsequent diagnosis of ATTR-CM established may relate to several factors. Our local practice has been to support early access to ^99m^Tc-DPD scintigraphy for patients in whom there is a high clinical suspicion for ATTR-CM, in advance of performing CMR. While CMR undoubtedly provides robust prognostic information (and should be performed in all patients with possible light chain cardiac amyloidosis), it is not a prerequisite for the diagnosis of ATTR-CM. All patients included in this study had echocardiography that was suggestive for cardiac amyloidosis prior to undergoing ^99m^Tc-DPD scintigraphy. The frequency of ‘false positives’ generated from echocardiography reflects its relative lack of specificity for the diagnosis of cardiac amyloidosis. Nonetheless, echocardiography is a crucial first-line investigation in simultaneously assessing systolic and diastolic function and plays a vital role in many cases in raising an initial suspicion of cardiac amyloidosis. The 45% rate of positive ^99m^Tc-DPD scintigraphy downstream from echocardiography findings that were deemed suggestive for amyloidosis in this study is acceptable, acknowledging the potential costs of missing a timely diagnosis of ATTR-CM.

There were, however, some important differences between the ATTR-CM and nonamyloid CM cohorts on baseline echocardiographic assessment. Relative to ATTR-CM, those patients with nonamyloid CM had lesser degrees of elevated LV wall thickness, LV mass and relative wall thickness; higher LV end-diastolic volumes, stroke volumes and myocardial contraction fraction; and smaller left atrial volumes. While patients with nonamyloid CM exhibited marginally higher GLS relative to those with ATTR-CM, GLS and the presence of apical sparing is not always discriminatory. Apical sparing of longitudinal strain once thought to be unique to cardiac amyloidosis,^29^^,^^30^ has now been shown to be present in aortic stenosis and also has a lower specificity in chronic kidney disease.^31^^,^^32^

Of the patients with nonamyloid CM those patients with uraemic CM were younger, more likely diabetic, and had the greatest elevation in cardiac biomarkers. Furthermore, patients with uraemic CM had the largest LV volumes (both in end-diastole and end-systole) stroke volume and myocardial volumes. Patients with hypertensive and uraemic CM also had high rates of diabetes mellitus which likely contributed to adverse LV remodelling.^33^ As expected, those patients with hypertrophic cardiomyopathy relative to other nonamyloid CM aetiologies had the greatest septal wall thickness (IVSd, mean 18 mm) and highest LV ejection fraction (mean 55%). Patients with non-amyloid CM had similar degrees of elevation in E/e’ across all aetiologies. Although there were some statistically significant differences in echocardiographic parameters at a disease cohort level, in this real-world study on an individual patient level, there was considerable overlap with very few clear definitive echocardiographic differentiators of pathology. There were no significant differences on baseline ECG in the frequency of atrial fibrillation between ATTR-CM, uraemic CM, hypertensive CM and aortic stenosis. In summary, patients with ATTR-CM, hypertensive CM, uraemic CM, HCM, and aortic stenosis can all appear strikingly similar, with considerable overlap in terms of demographics, ECG and echocardiographic findings. This observation confirms the critical role of ^99m^Tc-DPD scintigraphy in the diagnostic pathway for ATTR-CM.

Additional information obtained from CMR, using tissue characterization and multiparametric mapping enables CMR to help differentiate cardiac amyloidosis from other causes of a hypertrophic phenotype. Despite this, there is still commonality in echo and CMR imaging appearances for the remaining aetiologies responsible for a hypertrophic, restrictive phenotype. As technology advances, greater integration of artificial intelligence-enabled ECG and echocardiography readouts will undoubtedly help streamline the diagnostic process.^34^

### Study Limitations

This study is limited by the nature of its single centre, retrospective design with relatively small numbers of patients included between 2010 and 2019. We acknowledge the difficulty in assigning a single aetiology behind all forms of RCM; accepting the heterogeneous nature of the disease, this approach may represent an oversimiplification. While all patients with a confirmed diagnosis of ATTR-CM underwent *TTR* sequencing, not all patients with nonamyloid forms of RCM underwent genotyping. Finally, although not every patient underwent CMR, all patients were discussed in a video multidisciplinary team meeting that received input from clinicians based in centres with more than 20 years of expertise in amyloidosis and inherited heart muscle disease.

## Conclusions

In summary, the diagnosis of ATTR-CM is increasing driven by the emergence of novel targeted therapies which has led to a surge in the demand for ^99m^Tc-DPD scintigraphy over the last 15 years. As an unintended consequence, a cohort of patients with negative ^99m^Tc-DPD scintigraphy and alternative restrictive forms of HFpEF has emerged with a strikingly high mortality, equivalent to that of patients with ATTR-CM. There is a clear unmet need for further studies to better characterise this population, with a view to developing more targeted therapies and improve outcomes in this increasingly prevalent but seemingly overlooked cohort.

Perspectives **COMPETENCY IN MEDICAL KNOWLEDGE:** There is a high burden of mortality among patients referred for DPD scintigraphy in whom ATTR-CM is a differential diagnosis, irrespective of the imaging result. A greater awareness of the high mortality associated with nonamyloid forms of restrictive cardiomyopathy as well as ATTR-CM should prompt early consideration of pharmacological therapies such as mineralocorticoid receptor antagonists and SGLT2 inhibitors.

**TRANSLATIONAL OUTLOOK:** This study highlights the need to detect and diagnose nonamyloid forms of restrictive cardiomyopathy early, unravel the etiology, and develop targeted therapeutic agents to address the high mortality rate, which is equivalent to that of ATTR-CM.

## Funding support and author disclosures

Dr Moody has received advisory board fees from Alnylam, AstraZeneca, Bayer, BMS, Ionis Pharmaceuticals (formerly Akcea) and Pfizer. Dr Fontana has served as a consultant, or is on the advisory boards for Alnylam, Alexion/Caelum Biosciences, AstraZeneca, BridgeBio/Eidos, Prothena, Attralus, Intellia Therapeutics, Ionis Pharmaceuticals, Cardior, Lexeo Therapeutics, Janssen Pharmaceuticals, Pfizer and Novonordisk; has received research grants from Alnylam, BridgeBio, 10.13039/100004325AstraZeneca and 10.13039/100004319Pfizer; and has received a salary from the British Heart Foundation Intermediate Fellowship. Dr Gillmore has received research support from 10.13039/100006400Alnylam Pharmaceuticals; has received consulting fees from Alnylam Pharmaceuticals, AstraZeneca, BridgeBio, Intellia Therapeutics, Ionis Pharmaceuticals and Lycia Therapeutics; and has received payment for lectures or speaker fees from Alnylam Pharmaceuticals and AstraZeneca. All other authors have reported that they have no relationships relevant to the contents of this paper to disclose.
