# Supplementary material for: Impact of Increasing Referral for 99mTc-DPD Scintigraphy on Prognosis Across the Phenotypic Spectrum of Restrictive Cardiomyopathy
Source: JACC Adv. 2025 Oct 22;4(12):102230. doi: 10.1016/j.jacadv.2025.102230 (PMC12805169; doi:10.1016/j.jacadv.2025.102230)
Supplement: Supplemental_Material [file mmc1.pdf]

Supplementary material

(1) - Methods

Genotyping

All patients with confirmed ATTR-CM underwent TTR gene sequencing after informed written consent. DNA was extracted from whole blood and amplified by polymerase chain reaction assay, and the entire coding region of the TTR gene was sequenced by Sanger method. [(1), (2)]

Amyloid histology and immunohistochemistry

The presence of amyloid was demonstrated by the staining of amorphous material with Congo red that displayed apple-green birefringence when viewed under high-intensity cross-polarised light. Immunohistochemical staining of the amyloid deposits was performed using monospecific antibodies reactive with serum amyloid A protein, transthyretin, and with kappa and lambda immunoglobulin light chains.[(3)]

(2) Results

Supplemental Table 1. Baseline medical and device therapy.

**Variable**	**ATTRwt-CM (n=186)**	**ATTRv-CM (n=30)**	**ATTR-CM (n=216)**	**Non-amyloid cardiomyopathy (n=261)**	**p-value[d]**	**p-value[e]**
Anticoagulation, n (%)	139 (75%)	11 (37%)	150 (69%)	115 (44%)	<0.001	<0.001
Beta-blockers, n (%)	91 (49%)	8 (27%)	99 (46%)	154 (59%)	0.004	0.001
ACEi/Entresto, n (%)	106 (57%)	12 (40%)	118 (55%)	138 (53%)	0.70	0.21
MRA, n (%)	84 (45%)	18 (60%)	102 (47%)	64 (25%)	<0.001	<0.001
Digoxin, n (%)	46 (25%)	2 (7%)	48 (22%)	42 (16%)	0.088	0.015
SGLT2 inhibitor, n (%)	35 (19%)	6 (20%)	41 (19%)	23 (9%)	0.001	0.005
Phase 3 Trial, n (%)	27 (15%)	3 (10%)	30 (14%)	0 (0%)	<0.001	<0.001
Device, n (%)<br>None<br>VVI/DDD PPM<br>CRT<br>ICD<br>ILR<br>Missing data	 139 (75%)<br>22 (12%)<br>13 (7%)<br>4 (2%)<br>0 (0%)<br>8 (4%)	 24 (80%)<br>2 (7%)<br>2 (7%)<br>1 (3%)<br>1 (3%)<br>0 (0%)	 163 (75%)<br>24 (11%)<br>15 (7%)<br>5 (2%)<br>1 (1%)<br>8 (4%)	 194 (74%)<br>33 (13%)<br>10 (4%)<br>11 (4%)<br>3 (1%)<br>10 (4%)	 0.36	 0.39

Data are presented as n (%).

[d] For comparison between ATTR-CM and non-amyloid cardiomyopathy.

[e] For comparison between ATTRwt-CM, ATTRv-CM and non-amyloid cardiomyopathy.

Supplemental Table 2. Mortality rates among patients with non-amyloid cardiomyopathy according to diagnosis (n=206).

Variable	Hypertensive cardiomyopathy	Aortic stenosis	Hypertrophic cardiomyopathy	Uraemic cardiomyopathy
n	91	67	28	20
Mortality at follow up	25 (27.5%)	19 (28.4%)	8 (28.6%)	9 (45.0%)
Median follow up days [IQR]	943 [459 to 1505]	838 [495 to 1148]	1195 [524 to 1825]	1003 [529 to 1447]
Mean follow up days (SD)	997±581	844±450	1132±612	981±577
Total person-years of follow up	248.6	162.3	86.8	53.8
Mortality rate by person-years	0.10	0.12	0.09	0.17

Supplemental Table 3. Mortality rates among patients with transthyretin amyloid and non-amyloid cardiomyopathy according to diagnosis (n=477).

Variable	ATTRwt-CM	ATTRv-CM	Non-amyloid cardiomyopathy
n	186	30	261
Mortality at follow up	76 (40.9%)	11 (36.7%)	82 (31.4%)
Median follow up days [IQR]	780 [407 to 1172]	694 [493 to 1232]	915 [473 to 1377]
Mean follow up days (SD)	849±499	886±553	944±553
Total person-years of follow up	432.6	72.8	675.0
Mortality rate by person-years	0.176	0.151	0.121

References

1. Gillmore JD, Maurer MS, Falk RH, Merlini G, Damy T, Dispenzieri A, et al. Nonbiopsy Diagnosis of Cardiac Transthyretin Amyloidosis. Circulation. 2016;133(24):2404-12.

2. Sikora JL, Logue MW, Chan GG, Spencer BH, Prokaeva TB, Baldwin CT, et al. Genetic variation of the transthyretin gene in wild-type transthyretin amyloidosis (ATTRwt). Hum Genet. 2015;134(1):111-21.

3. Puchtler H, Sweat F, Levine M. On the binding of Congo red by amyloid. Journal of Histochemistry & Cytochemistry. 1962;10(3):355-64.

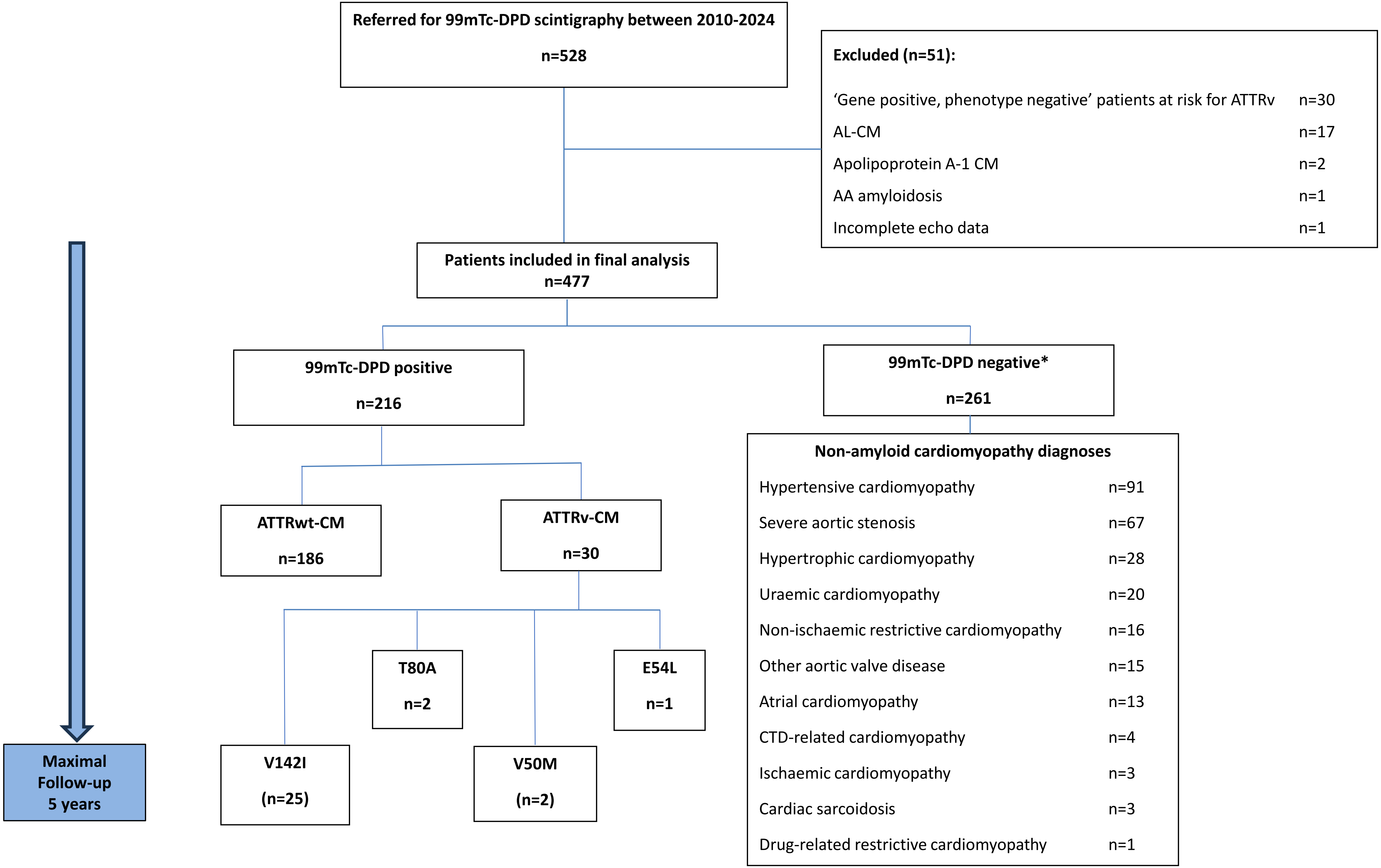
Referred for 99mTc-DPD scintigraphy between 2010-2024
n=528
Excluded (n=51):
'Gene positive, phenotype negative' patients at risk for ATTRv n=30
AL-CM n=17
Apolipoprotein A-1 CM n=2
AA amyloidosis n=1
Incomplete echo data n=1
Patients included in final analysis
n=477
99mTc-DPD positive
n=216
99mTc-DPD negative*
n=261
Non-amyloid cardiomyopathy diagnoses
Hypertensive cardiomyopathy n=91
Severe aortic stenosis n=67
Hypertrophic cardiomyopathy n=28
Uraemic cardiomyopathy n=20
Non-ischaemic restrictive cardiomyopathy n=16
Other aortic valve disease n=15
Atrial cardiomyopathy n=13
CTD-related cardiomyopathy n=4
Ischaemic cardiomyopathy n=3
Cardiac sarcoidosis n=3
Drug-related restrictive cardiomyopathy n=1
ATTRwt-CM
n=186
ATTRv-CM
n=30
T80A
n=2
E54L
n=1
V142I
(n=25)
V50M
(n=2)
Maximal
Follow-up
5 years

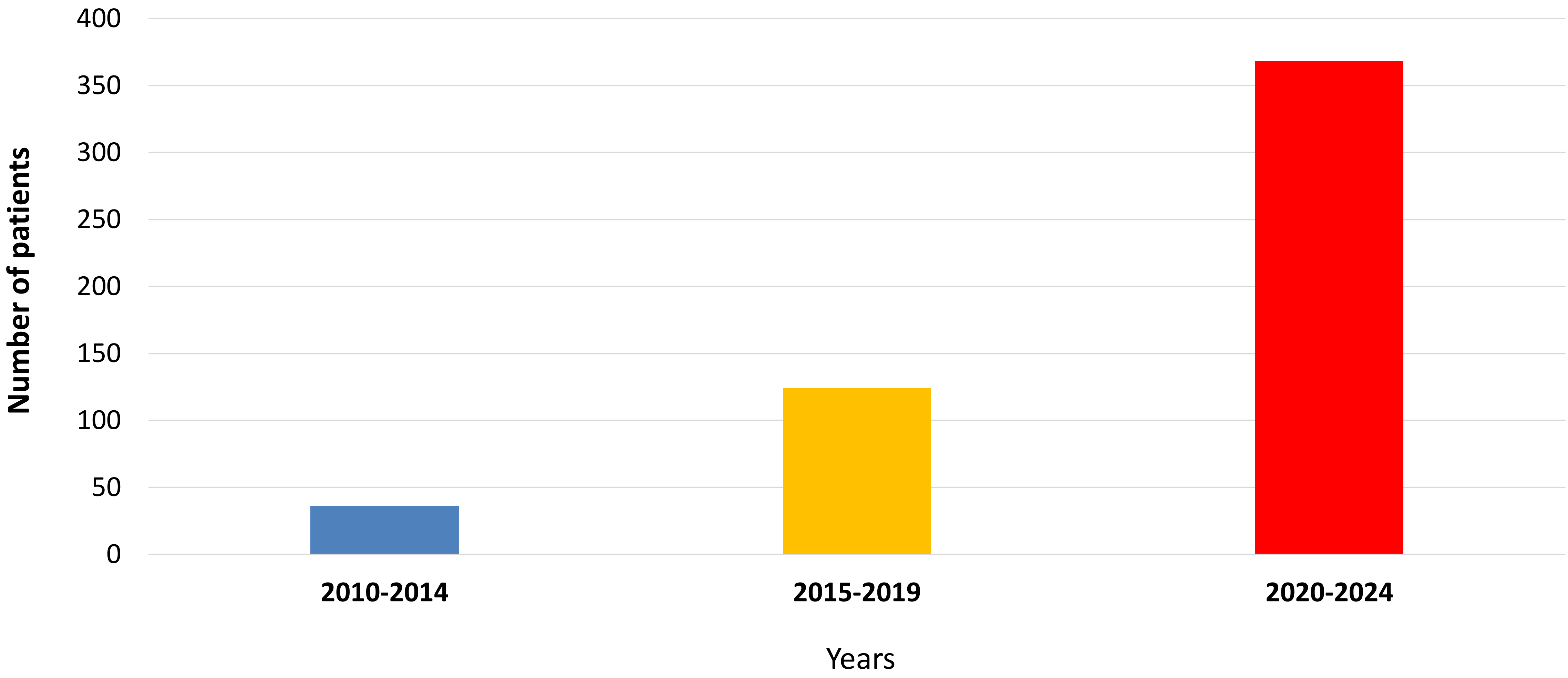

Number of patients
400
350
300
250
200
150
100
50
0
2010-2014
2015-2019
2020-2024
Years

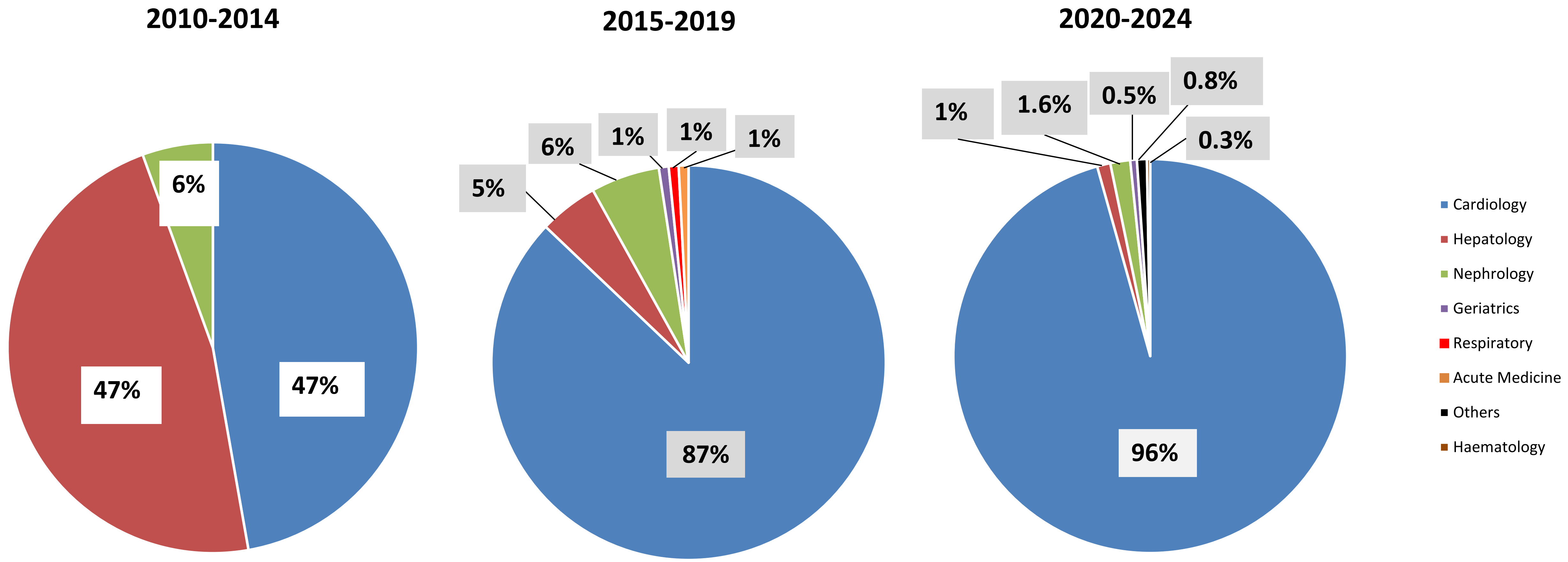
2010-2014
6%
47%
47%
2015-2019
5%
6%
1%
1%
1%
87%
2020-2024
1%
1.6%
0.5%
0.8%
0.3%
96%
Cardiology
Hepatology
Nephrology
Geriatrics
Respiratory
Acute Medicine
Others
Haematology

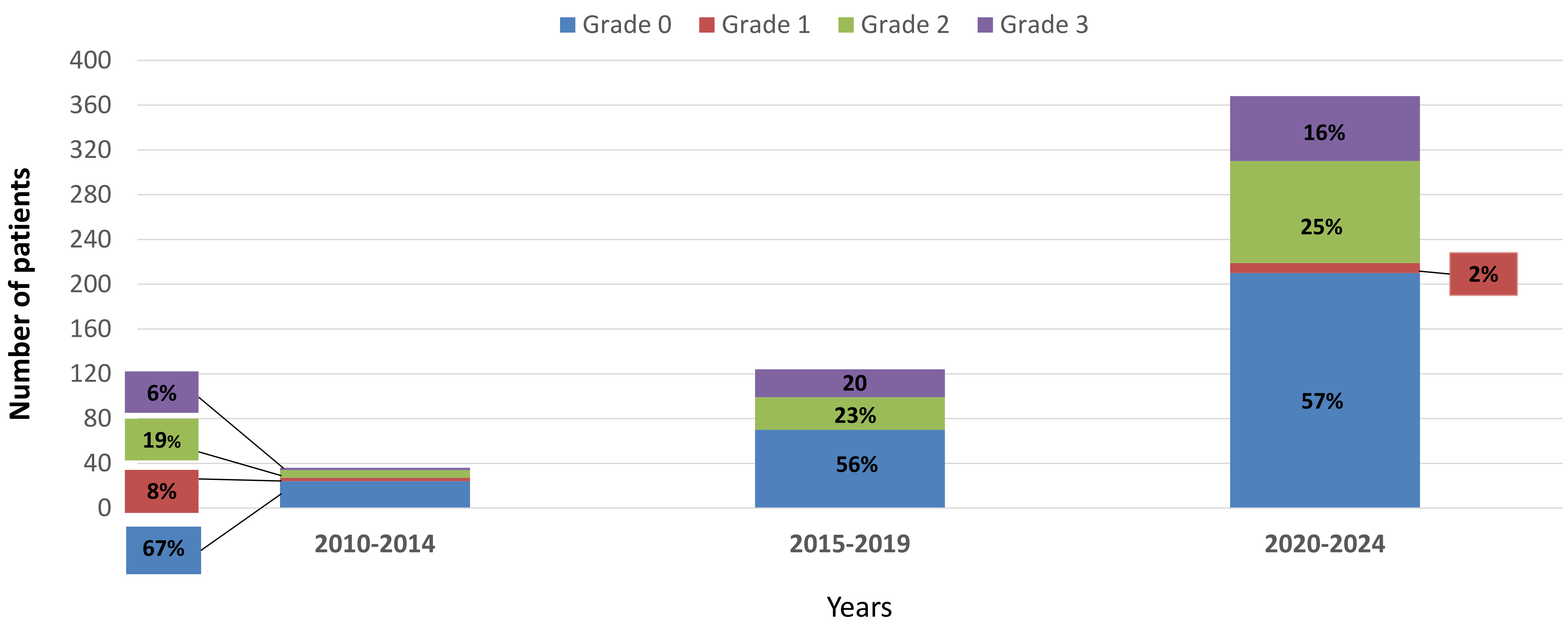
Grade 0
Grade 1
Grade 2
Grade 3
Number of patients
400
360
320
280
240
200
160
120
80
40
0
6%
19%
8%
67%
20
23%
56%
16%
25%
2%
57%
2010-2014
2015-2019
2020-2024
Years

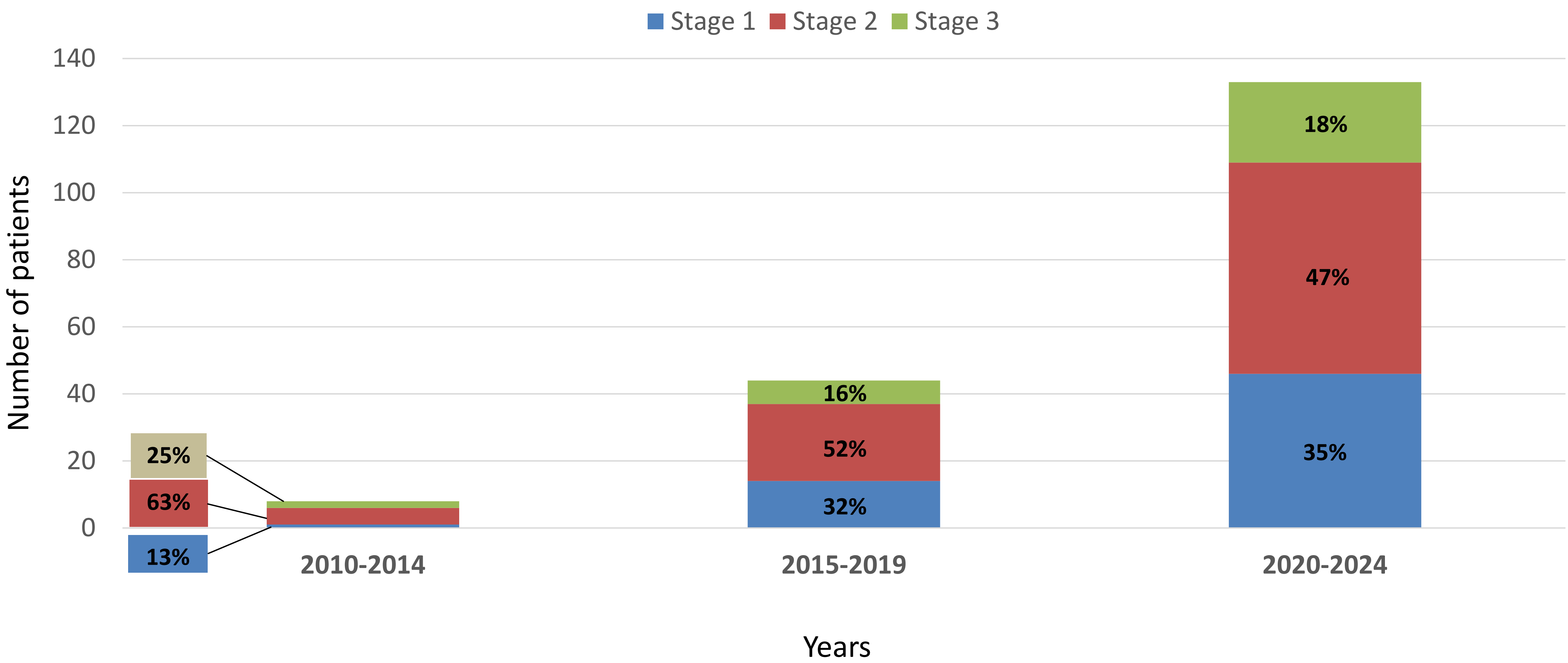

Stage 1
Stage 2
Stage 3
Number of patients
140
120
100
80
60
40
20
0
25%
63%
13%
16%
52%
32%
18%
47%
35%
2010-2014
2015-2019
2020-2024
Years

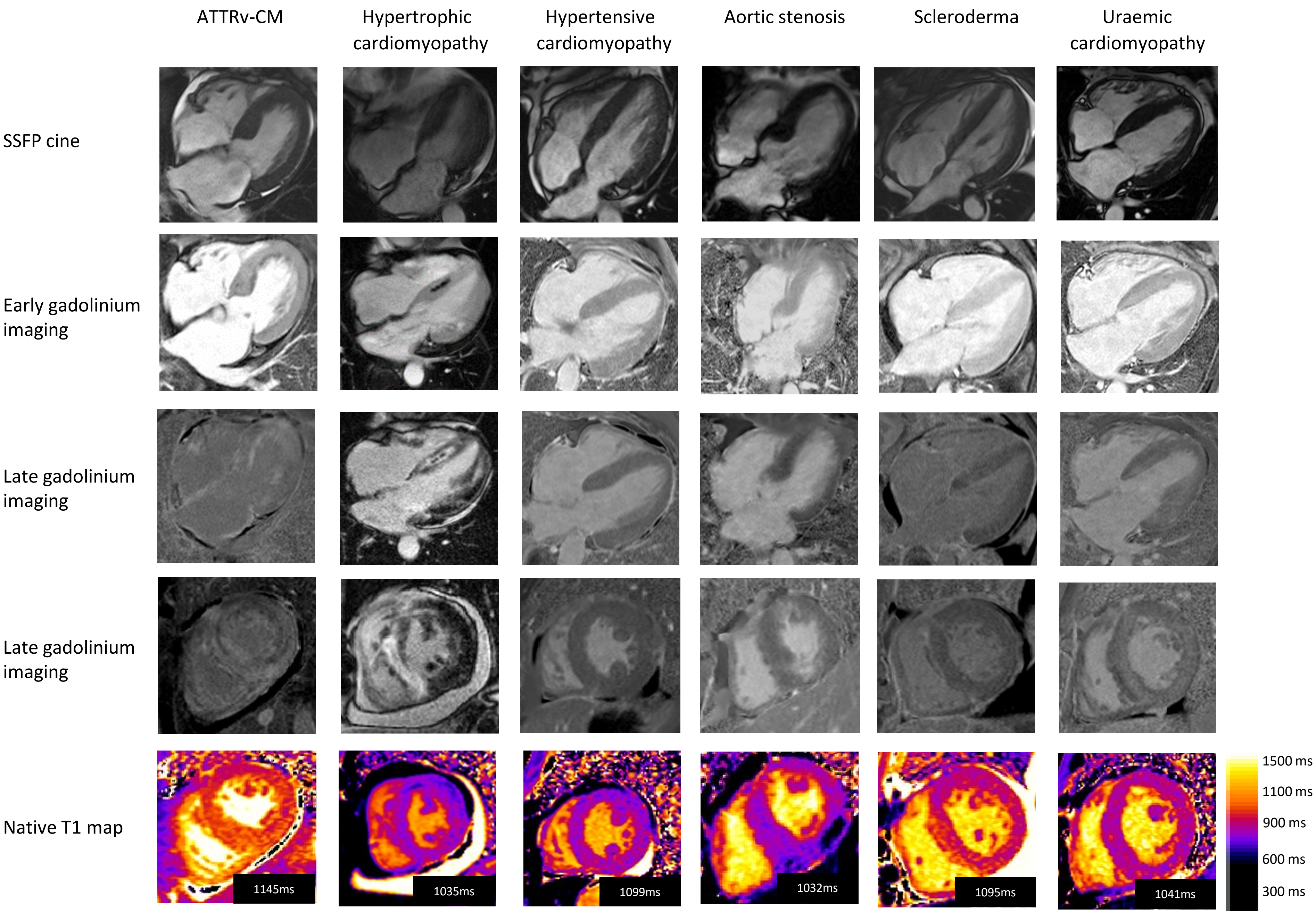

ATTRv-CM
Hypertrophic cardiomyopathy
Hypertensive cardiomyopathy
Aortic stenosis
Scleroderma
Uraemic cardiomyopathy
SSFP cine
Early gadolinium imaging
Late gadolinium imaging
Late gadolinium imaging
Native T1 map
1145ms
1035ms
1099ms
1032ms
1095ms
1041ms
1500 ms
1100 ms
900 ms
600 ms
300 ms

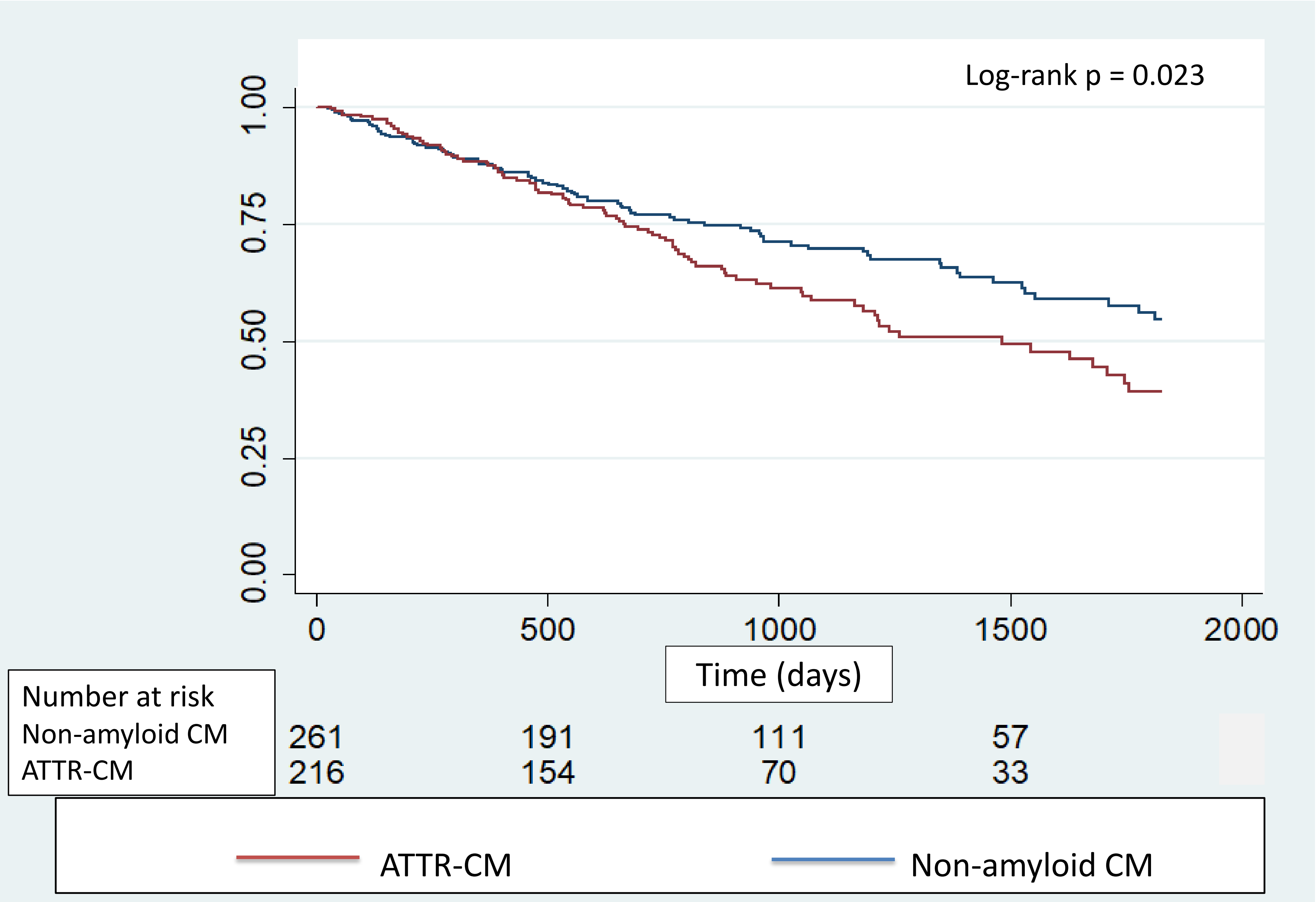

Log-rank p = 0.023
1.00
0.75
0.50
0.25
0.00
0
500
1000
1500
2000
Time (days)
Number at risk
Non-amyloid CM
ATTR-CM
261
191
111
57
216
154
70
33
ATTR-CM
Non-amyloid CM

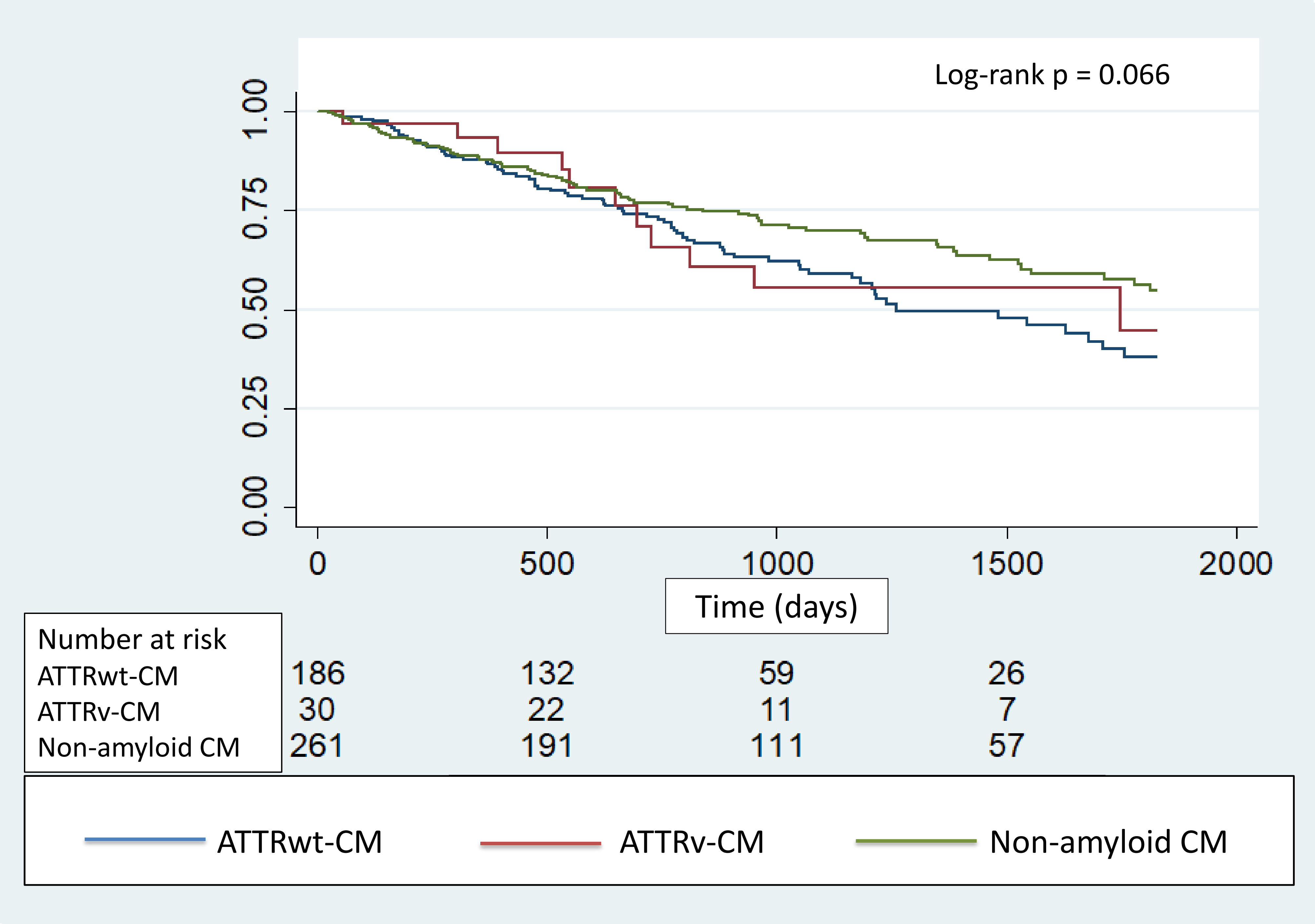
Log-rank p = 0.066
1.00
0.75
0.50
0.25
0.00
0
500
1000
1500
2000
Time (days)
Number at risk
ATTRwt-CM 186 132 59 26
ATTRv-CM 30 22 11 7
Non-amyloid CM 261 191 111 57
ATTRwt-CM
ATTRv-CM
Non-amyloid CM

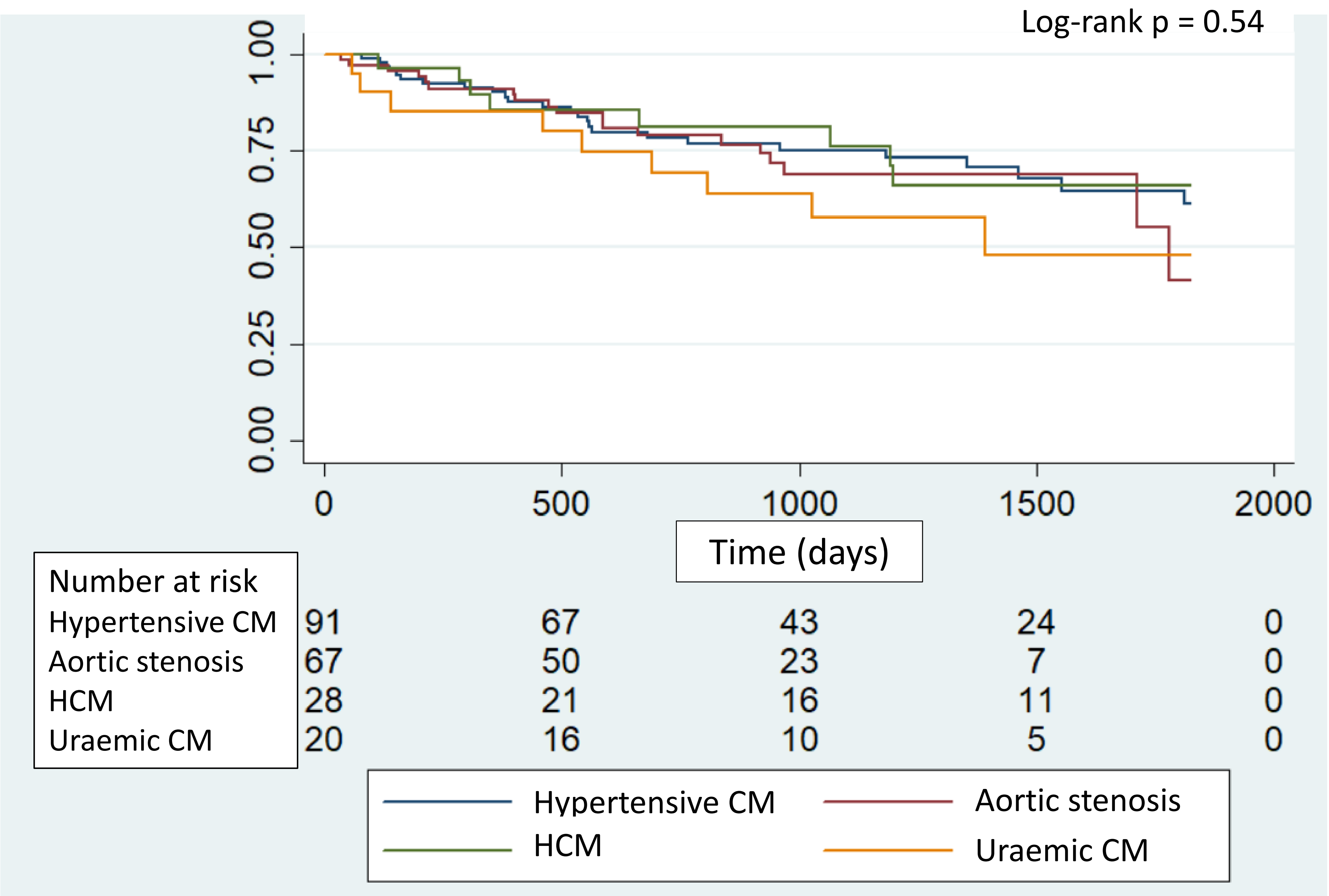

Log-rank p = 0.54
0.00
0.25
0.50
0.75
1.00
0
500
1000
1500
2000
Time (days)
Number at risk
Hypertensive CM 91 67 43 24 0
Aortic stenosis 67 50 23 7 0
HCM 28 21 16 11 0
Uraemic CM 20 16 10 5 0
Hypertensive CM
Aortic stenosis
HCM
Uraemic CM

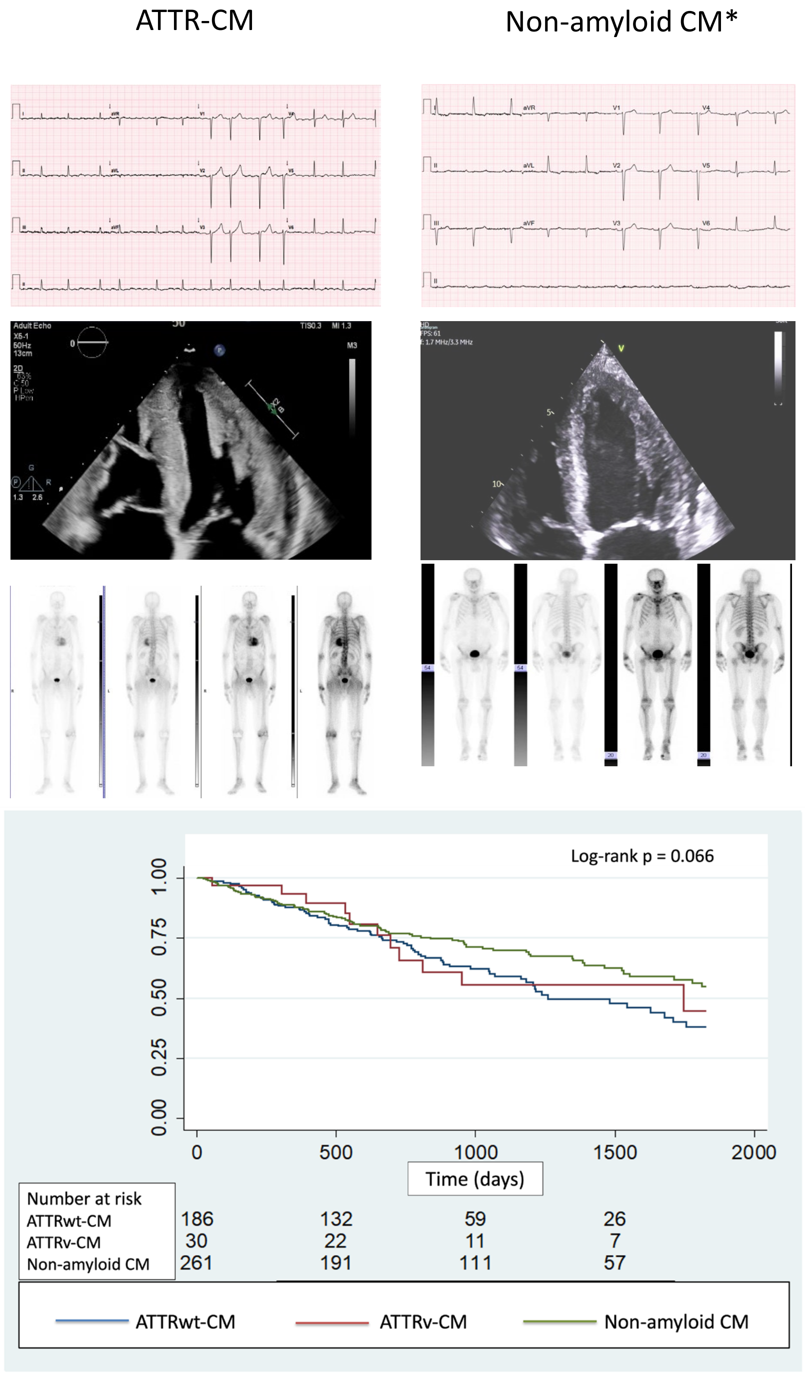

ATTR-CM
Non-amyloid CM*
Log-rank p = 0.066
0.00
0.25
0.50
0.75
1.00
0
500
1000
1500
2000
Time (days)
Number at risk
ATTRwt-CM 186 132 59 26
ATTRv-CM 30 22 11 7
Non-amyloid CM 261 191 111 57
ATTRwt-CM
ATTRv-CM
Non-amyloid CM

STROBE Statement—checklist of items that should be included in reports of observational studies

	Item No.	Recommendation	Page No.	Relevant text from manuscript
**Title and abstract**	1	(*a*) Indicate the study's design with a commonly used term in the title or the abstract	1	Impact of increasing referral...
		(*b*) Provide in the abstract an informative and balanced summary of what was done and what was found	3	
**Introduction**				
Background/rationale	2	Explain the scientific background and rationale for the investigation being reported	5	
Objectives	3	State specific objectives, including any prespecified hypotheses	6	The aims of the present study...
**Methods**				
Study design	4	Present key elements of study design early in the paper	7	Retrospective, cohort study
Setting	5	Describe the setting, locations, and relevant dates, including periods of recruitment, exposure, follow-up, and data collection	7	Single-centre; January 2014-August 2023
Participants	6	(*a*) *Cohort study*—Give the eligibility criteria, and the sources and methods of selection of participants. Describe methods of follow-up<br>*Case-control study*—Give the eligibility criteria, and the sources and methods of case ascertainment and control selection. Give the rationale for the choice of cases and controls<br>*Cross-sectional study*—Give the eligibility criteria, and the sources and methods of selection of participants	7	
		(*b*) *Cohort study*—For matched studies, give matching criteria and number of exposed and unexposed<br>*Case-control study*—For matched studies, give matching criteria and the number of controls per case		
Variables	7	Clearly define all outcomes, exposures, predictors, potential confounders, and effect modifiers. Give diagnostic criteria, if applicable	7-10, supplementary material	
Data sources/ measurement	8*	For each variable of interest, give sources of data and details of methods of assessment (measurement). Describe comparability of assessment methods if there is more than one group	7-10	
Bias	9	Describe any efforts to address potential sources of bias	10	
Study size	10	Explain how the study size was arrived at	7	

Continued on next page

Quantitative variables	11	Explain how quantitative variables were handled in the analyses. If applicable, describe which groupings were chosen and why	10
Statistical methods	12	(*a*) Describe all statistical methods, including those used to control for confounding	10
		(*b*) Describe any methods used to examine subgroups and interactions	10
		(*c*) Explain how missing data were addressed	
		(*d*) *Cohort study*—If applicable, explain how loss to follow-up was addressed<br>*Case-control study*—If applicable, explain how matching of cases and controls was addressed<br>*Cross-sectional study*—If applicable, describe analytical methods taking account of sampling strategy	10
		(*e*) Describe any sensitivity analyses	10
**Results**			
Participants	13*	(a) Report numbers of individuals at each stage of study—eg numbers potentially eligible, examined for eligibility, confirmed eligible, included in the study, completing follow-up, and analysed	11
		(b) Give reasons for non-participation at each stage	11
		(c) Consider use of a flow diagram	11, Figure 1
Descriptive data	14*	(a) Give characteristics of study participants (eg demographic, clinical, social) and information on exposures and potential confounders	11-15
		(b) Indicate number of participants with missing data for each variable of interest	31-40
		(c) *Cohort study*—Summarise follow-up time (eg, average and total amount)	11
Outcome data	15*	*Cohort study*—Report numbers of outcome events or summary measures over time	11-16
		Case-control study—Report numbers in each exposure category, or summary measures of exposure	
		Cross-sectional study—Report numbers of outcome events or summary measures	
Main results	16	(*a*) Give unadjusted estimates and, if applicable, confounder-adjusted estimates and their precision (eg, 95% confidence interval). Make clear which confounders were adjusted for and why they were included	11-16
		(*b*) Report category boundaries when continuous variables were categorized	11-16
		(*c*) If relevant, consider translating estimates of relative risk into absolute risk for a meaningful time period	

Continued on next page

Other analyses	17	Report other analyses done—eg analyses of subgroups and interactions, and sensitivity analyses	15
**Discussion**			
Key results	18	Summarise key results with reference to study objectives	17
Limitations	19	Discuss limitations of the study, taking into account sources of potential bias or imprecision. Discuss both direction and magnitude of any potential bias	21-22
Interpretation	20	Give a cautious overall interpretation of results considering objectives, limitations, multiplicity of analyses, results from similar studies, and other relevant evidence	17-22
Generalisability	21	Discuss the generalisability (external validity) of the study results	17-22
**Other information**			
Funding	22	Give the source of funding and the role of the funders for the present study and, if applicable, for the original study on which the present article is based	1, 23

*Give information separately for cases and controls in case-control studies and, if applicable, for exposed and unexposed groups in cohort and cross-sectional studies.

Note: An Explanation and Elaboration article discusses each checklist item and gives methodological background and published examples of transparent reporting. The STROBE checklist is best used in conjunction with this article (freely available on the Web sites of PLoS Medicine at http://www.plosmedicine.org/, Annals of Internal Medicine at http://www.annals.org/, and Epidemiology at http://www.epidem.com/). Information on the STROBE Initiative is available at www.strobe-statement.org.
